# FOXA2 suppresses endometrial carcinogenesis and epithelial-mesenchymal transition by regulating enhancer activity

**DOI:** 10.1172/JCI157574

**Published:** 2022-06-15

**Authors:** Subhransu S. Sahoo, Susmita G. Ramanand, Yunpeng Gao, Ahmed Abbas, Ashwani Kumar, Ileana C. Cuevas, Hao-Dong Li, Mitzi Aguilar, Chao Xing, Ram S. Mani, Diego H. Castrillon

**Affiliations:** 1 Department of Pathology,; 2 Eugene McDermott Center for Human Growth and Development,; 3 Department of Bioinformatics,; 4 Department of Population and Data Sciences,; 5 Harold C. Simmons Comprehensive Cancer Center,; 6 Department of Urology, and; 7 Department of Obstetrics and Gynecology, UT Southwestern Medical Center, Dallas, Texas, USA.

**Keywords:** Oncology, Cancer, Molecular pathology, Tumor suppressors

## Abstract

*FOXA2* encodes a transcription factor mutated in 10% of endometrial cancers (ECs), with a higher mutation rate in aggressive variants. FOXA2 has essential roles in embryonic and uterine development. However, FOXA2’s role in EC is incompletely understood. Functional investigations using human and mouse EC cell lines revealed that FOXA2 controls endometrial epithelial gene expression programs regulating cell proliferation, adhesion, and endometrial-epithelial transition. In live animals, conditional inactivation of *Foxa2* or *Pten* alone in endometrial epithelium did not result in ECs, but simultaneous inactivation of both genes resulted in lethal ECs with complete penetrance, establishing potent synergism between Foxa2 and PI3K signaling. Studies in tumor-derived cell lines and organoids highlighted additional invasion and cell growth phenotypes associated with malignant transformation and identified key mediators, including *Myc* and *Cdh1*. Transcriptome and cistrome analyses revealed that FOXA2 broadly controls gene expression programs through modification of enhancer activity in addition to regulating specific target genes, rationalizing its tumor suppressor functions. By integrating results from our cell lines, organoids, animal models, and patient data, our findings demonstrated that FOXA2 is an endometrial tumor suppressor associated with aggressive disease and with shared commonalities among its roles in endometrial function and carcinogenesis.

## Graphical abstract

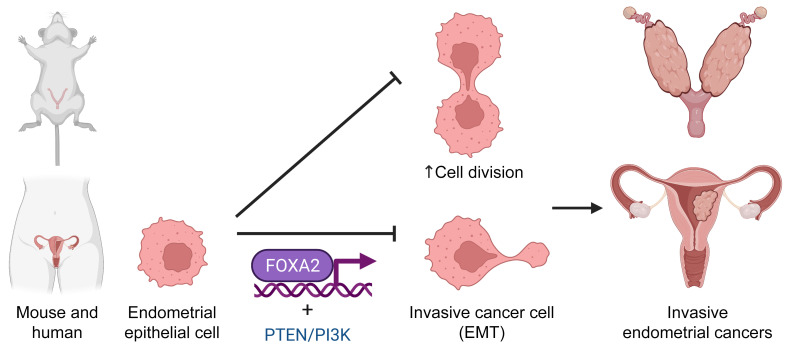

## Introduction

FOXA1 and FOXA2 are winged-helix pioneer transcription factors that are essential cell type–specific regulators of organogenesis (1). They were initially identified through biochemical approaches as hepatocyte-enriched HNF-3 DNA-binding proteins (2) and subsequently found to be homologous to *Drosophila* forkhead (3). Unlike FOXOs, a distinct FOX subfamily regulated by AKT-mediated phosphorylation leading to FOXO cytoplasmic sequestration and inactivation (4–7), FOXA1/2 are constitutively nuclear (1, 8). FOXA1/2 are highly expressed in the embryonic endoderm and epithelial lining of diverse organs derived from the endoderm, including the digestive, respiratory, and reproductive tracts. A third member of the FOXA family (FOXA3) shares less similarity and has more restricted patterns of expression (9). *FOXA3*-KO mice have normal lifespan without a tumor-prone phenotype (10), and *FOXA3* mutations have not been reported in human cancer. *FOXA1* and *FOXA2* have partially redundant functions during embryogenesis. For example, mouse embryos deficient for either one undergo normal liver specification, whereas in doubly deficient embryos, hepatic specification is blocked (11). Similarly, branching epithelial morphogenesis in the pancreas and lung is dependent upon the joint action of *FOXA1/2* (12, 13). On the other hand, the extent of functional redundancy among *FOXA1/2* appears to be tissue- or cell type–dependent and contingent at least in part on their spatiotemporal patterns of expression. For example, embryos null for *FOXA2* alone lack foregut endoderm and notochord linked to earlier induction of *FOXA2* relative to *FOXA1*, resulting in death by embryonic day 9 (1). During gastrulation, *FOXA2* suppresses epithelial-mesenchymal transition (EMT), preventing the endoderm from undergoing EMT (14).

In the developing mouse and human uterus, FOXA2 protein is expressed in the glandular but not surface epithelium, coincident with the initiation of budding gland formation (adenogenesis) postnatally (15–17). Mice with endometrium-specific *Foxa2* ablation fail to form endometrial glands with persistence of only the surface epithelium (18) (in accordance with nomenclature conventions, all capital letters will be used unless mouse Foxa2 is specifically denoted). Foxa2 is also essential for uterine function and fertility later in life and during pregnancy, as demonstrated in mouse models where *Foxa2* was ablated postnatally, permitting normal gland formation (19, 20). In the context of normal human uterine function, genome-wide mapping of *FOXA2* binding sites integrated with transcriptome studies have defined dynamic changes in gene regulation in the proliferative and secretory phases of the reproductive cycle and identified FOXA2-regulated genes that influence uterine receptivity and blastocyst implantation (19). Thus, these studies have shown that FOXA2 is essential for endometrial gland development during the maturation of the female reproductive tract, but also serves indispensable functions in endometrial function and embryo receptivity during adult life.

Strikingly, the endometrial cancer (EC) genome atlas project in The Cancer Genome Atlas (TCGA) discovered a significant *FOXA2* mutation rate among endometrioid adenocarcinomas, the major histologic subtype of EC. Mutations occurred across the coding region, suggesting that in the endometrium, *FOXA2* functions as a tumor suppressor where loss of activity drives tumorigenesis. Commonly recurring mutations in *FOXA1* were not identified (21). A subsequent study specifically evaluating *FOXA2* reported an endometrioid adenocarcinoma mutation rate of 9.4%, with a spectrum of mutations again interpreted as consistent with a tumor suppressor role (22). *FOXA2* mutations are even more frequent (15%) in uterine carcinosarcomas and clear cell carcinomas, 2 highly lethal EC subtypes (23). Other studies have confirmed recurring *FOXA2* somatic mutations in ECs (24). Among 32 TCGA data sets of diverse human cancers (cBioPortal), endometrial carcinomas and carcinosarcomas are the 2 cancer types with the highest incidence of *FOXA2* mutation, affirming that *FOXA2* plays a particularly important — and perhaps unique — role in endometrial carcinogenesis (25). Conversely, in prostate cancer, *FOXA2* mutations are rare/absent, whereas *FOXA1* mutations are common, with diverse experimental evidence that different classes of *FOXA1* mutations in prostate cancer are dominant and gain of function. In other words, *FOXA1* is an oncogene in prostate cancer (26–33), whereas in EC, the evidence has suggested that *FOXA2* is a tumor suppressor.

Although one study employed EC cell lines to show that engineered *FOXA2* mutations have variable effects on transcription factor activity (34), the mechanisms by which *FOXA2* mutations promote endometrial carcinogenesis remain largely undefined, in part due to the lack of genetic models faithfully recapitulating *FOXA2*-driven endometrial tumorigenesis. In this study, we created a genetically engineered model of *FOXA2*-deficient EC and used it in combination with human and mouse cancer cell line systems to address diverse critical questions relating to the biological roles of FOXA2 in EC initiation and progression. These formal genetic and functional analyses demonstrated that FOXA2 functions as a multitasking tumor suppressor controlling diverse cell growth and adhesion pathways through its transcriptional pioneering activity, rationalizing *FOXA2* inactivating mutations as potent drivers of EC and providing conceptual linkages to its known roles in embryonic development.

## Results

### Inverse patterns of FOXA1 and FOXA2 expression in prostate and endometrial epithelium rationalize their specific roles as prostate versus EC drivers.

**Figure 1:**
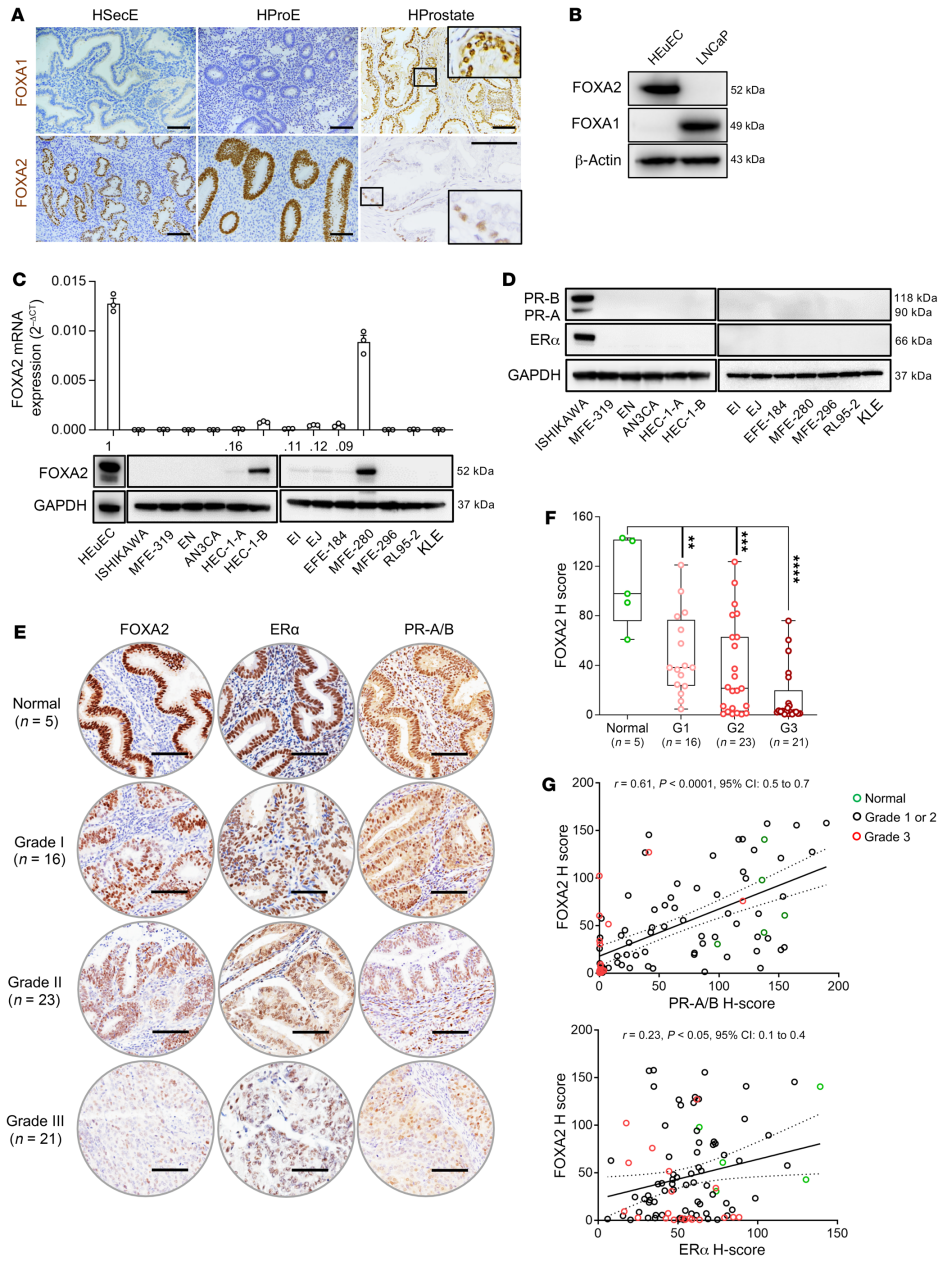
FOXA2 is the endometrium-specific FOXA and is downregulated in ECs. (**A**) Tissue sections of normal human endometrium (secretory/HSecE, proliferative/HProE) and prostate (HProstate) immunostained for FOXA1 or FOXA2. FOXA1 is expressed in the prostate (luminal/secretory and basal layers) but not endometrium, whereas FOXA2 is expressed in endometrial epithelial cells and only weakly and heterogeneously in the basal layer of prostate glands. The 6 immunostains were performed under identical conditions, antibody concentrations, and chromogen incubation times and are thus directly comparable to one another. Insets for HProstate are higher magnifications of smaller boxed areas. Scale bars: 100 μm. (**B**) Western blot of immortalized endometrium (HEuEC) and prostate cancer (LNCaP) cell lines confirmed mutually exclusive patterns of FOXA1 and FOXA2 expression. (**C**) mRNA expression levels by qRT-PCR (*n =* 3) and correlation to protein expression by Western blot in 1 immortalized normal endometrial cell line (HEuEC) and 13 EC cell lines. FOXA2 was undetectable in 7/13 and significantly downregulated in 4/13 of the EC cell lines. Data shown as mean ± SEM. (**D**) Western blot analysis of estrogen receptor α (ERα) and progesterone receptor-A/B (PR-A/B) in EC cell lines. (**E**) Expression of FOXA2, ERα, and PR in normal human endometrium and grade 1–3 human endometrial carcinomas; representative images. Scale bars: 100 μm. (**F**) FOXA2 expression levels per H-scores in normal endometrium (*n =* 5) and ECs (grade 1, *n =* 16; grade 2, *n =* 23; grade 3, *n =* 21). Box-and-whisker plot represents medians with minimum and maximum values. *P* value was determined by 2-tailed Mann-Whitney *U* tests compared with normal cases. Data are shown as mean ± SEM; **P < 0.01, ***P < 0.001, ****P < 0.0001. (**G**) Scatter plot shows correlation analysis of FOXA2 with ERα and PR in normal endometrium and ECs. FOXA2, ERα, and PR expression levels were determined by H-score. Pearson’s *r* determined correlation between data groups.

The mutual exclusivity of *FOXA1* and *FOXA2* mutations in prostate cancer and EC might seem counterintuitive given that they share diverse embryonic functions and broad expression across the endoderm and its derivatives (1). To explore this question, we performed IHC in the normal human uterus and prostate. In normal endometrium, FOXA1 expression was undetectable in either the proliferative (preovulatory) or secretory (postovulatory) phases. In contrast, in the prostate (which unlike the endometrium has a bilayered epithelium), FOXA1 was strongly expressed in both the basal and luminal layers. In the endometrium, FOXA2 was strongly expressed in glandular epithelium in both the secretory and proliferative phases, consistent with prior studies (18, 19), whereas in the prostate, FOXA2 was expressed only in the basal layer and expression was weak and heterogenous (Figure 1A), consistent with a prior study of FOXA2 expression in the prostate (35). Then, we analyzed the expression of both factors in an immortalized (noncancerous) endometrial cell line (HEuEC) and a prostate cancer cell line (LNCaP) by Western blot. FOXA2 was expressed only in the endometrial cell line, while FOXA1 was expressed only in the prostate cell line (Figure 1B), in agreement with the tissue expression patterns and that most prostatic adenocarcinomas and LNCaP are of secretory (i.e., luminal) cell origin (36). Thus, the differential and highly specific occurrence of *FOXA1* or *FOXA2* mutations in prostate versus ECs is readily explained by (a) high expression of FOXA1 in the prostatic luminal epithelial layer, (b) FOXA1’s lack of expression in endometrium, and (c) the inverse patterns of FOXA2 expression in these cell types.

### Characterization of FOXA2 expression in human EC cell lines and primary tumors implicates loss of FOXA2 expression as a cancer driver.

First, we analyzed FOXA2 expression in a comprehensive panel of EC cell lines by quantitative reverse transcription PCR (qRT-PCR) and Western blot analysis. FOXA2 was highly expressed in HEuEC cells but absent or markedly decreased in the majority of EC cell lines (11/13, Figure 1C), consistent with previously reported findings for 7 of these cell lines (23). Per the Catalogue of Somatic Mutations in Cancer (COSMIC) and the Broad Institute Cancer Cell Line Encyclopedia, at least 3 of these cell lines have *FOXA2* mutations likely to be functionally significant (EN, p.Y170C; MFE-319, p.S449F and p.M424Cfs*37; and EFE-184, N214S) (37, 38). Nonetheless, the dramatic loss of FOXA2 expression in the majority of endometrial cell lines strongly suggests that (a) mutation rates underestimate the significance of *FOXA2* as an endometrial tumor suppressor, and (b) additional, i.e., epigenetic or other regulatory mechanisms may account for FOXA2 protein loss. To further characterize the expression of other endometrial differentiation markers and factors with essential roles in endometrial growth and differentiation, we analyzed the expression of the estrogen and progesterone receptors in the same cell line panel. Both estrogen receptor α (ERα) and progesterone receptor-A/B (PR-A/B) were highly expressed in the Ishikawa (ISK) cell line (Figure 1D), which for this reason is frequently used for studies of steroid hormone receptivity (39). However, expression was lost in all of the other cell lines (Figure 1D). Thus, FOXA2 was generally lost in EC cell lines, as occurred for both ERα and PR-A/B. Observations made in our mouse model (see below) suggest that although the loss of 3 factors was highly correlated, FOXA2 loss was not directly or immediately responsible for ERα and PR-A/B downregulation during EC progression.

To extend these findings to human primary ECs, expression of FOXA2, ERα, and PR were assessed in a tissue microarray of normal endometrium (*n =* 5) and grade 1 (*n =* 16), 2 (*n =* 23), and 3 (*n =* 21) endometrioid adenocarcinomas. After immunostaining using the validated monoclonal antibodies, expression intensity was determined by image analysis. Whereas robust expression was consistently observed in normal endometrium, expression of all 3 markers by image analysis (H-score) was significantly decreased or absent in a grade-dependent manner (Figure 1, E and F). The total number of cases expressing any FOXA2 (H-score >0) were the following: normal (5/5, 100%), G1 (16/16, 100%), G2 (18/23, 78%), and G3 (12/21, 57%). Furthermore, both ERα and PR expression levels positively correlated with FOXA2 expression levels in individual tumors (Figure 1G; ERα: Pearson’s correlation coefficient, *r* = 0.23, *P* < 5 × 10^–2^ and PR: *r* = 0.61, *P* < 1 × 10^–4^). These studies demonstrated that all 3 factors were consistently downregulated in ECs, and this downregulation depended upon tumor grade. These findings, while correlative, strongly suggest that FOXA2 is a classic tumor suppressor wherein loss or downregulation drives tumor progression.

### Enforced FOXA2 reexpression in a FOXA2-deficient EC cell line inhibits growth and invasive phenotypes through upregulation of cell adhesion factors.

**Figure 2:**
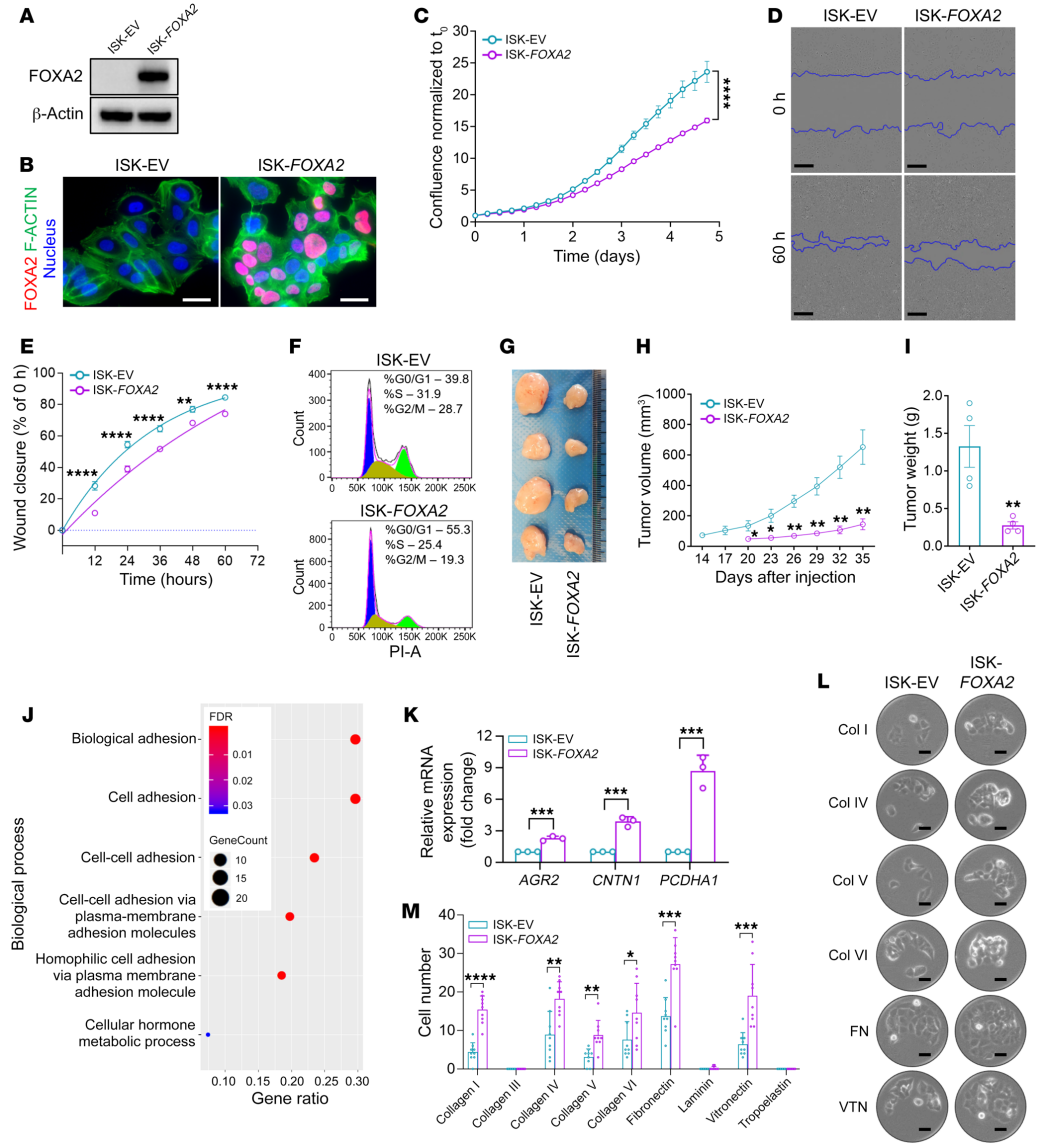
FOXA2 suppresses EC cell proliferation and enhances cell adhesion. (**A**) FOXA2 reexpression after lentiviral transduction in Ishikawa (ISK) cells (ISK-*FOXA2*). (**B**) Immunofluorescence shows expected FOXA2 nuclear localization, DAPI counterstain. Scale bars: 50 μm. (**C**) Cell growth analysis showing *FOXA2*-mediated growth suppression; confluency normalized to t_0_ (*n =* 3). Data shown as mean ± SEM; 2-tailed *t* test. (**D**) Wound-healing assay at t_0_ and 60 hours. Scale bars: 250 μm. (**E**) Wound closure per gap distance (*n =* 3). Data shown as mean ± SEM; 2-tailed *t* test. (**F**) Cell cycle analysis (*n =* 3). Peaks in blue, yellow, and green show percentage cells in G0/G1, S, and G2/M phase. (**G**) Xenografts after s.c. injection of 1 million cells in left/right flanks of NOD *scid* gamma females (*n =* 4). Tumors harvested 35 days after injection. (**H**) Growth curves of ISK-EV and ISK-*FOXA2* xenografts per caliper measurements (*n =* 4). Data shown as mean ± SEM; 2-tailed *t* test. (**I**) Endpoint xenograft weights at day 35 (*n =* 4, same tumors shown in **G**). Data shown as mean ± SEM; 2-tailed *t* test. (**J**) Gene Ontology (GO) enrichment analysis of 89 significantly upregulated genes by RNA-Seq (≥4-fold and *P <* 0.0001) in ISK-*FOXA2* cells. GO pathways plotted by gene ratio. Dots sized in proportion to gene numbers in the GO term colored by FDR value per inset. (**K**) Relative change in mRNA expression of *AGR2*, *CNTN1*, and *PCDHA1* genes (*n =* 3). Data shown as mean ± SEM; multiple 2-tailed *t* tests. (**L**) Cell adhesion assays, representative phase contrast images of ISK-EV and ISK-*FOXA2* cells adhering to extracellular matrix protein panel after 16 hours. Scale bars: 50 μm. (**M**) Quantitative analysis, cell adhesion assays (*n =* 9). Data shown as mean ± SEM; multiple 2-tailed *t* tests. For all panels, *P < 0.05; **P < 0.01; ***P < 0.001; ****P < 0.0001.

ISK cells, which are FOXA2 deficient, were transduced with a lentiviral vector harboring a full-length *FOXA2* cDNA (ISK-*FOXA2*). For controls, cells were transfected with empty vector (ISK-EV). After drug selection, Western blotting confirmed stable reexpression of the FOXA2 protein, and immunofluorescence of the cultured cells confirmed the expected nuclear localization of FOXA2 (Figure 2, A and B). Comparison of the 2 isogenic cell lines by standard growth assays showed that FOXA2 reexpression resulted in a significant decrease in cell proliferation by 5 days in culture (Figure 2C); similar results were observed with an additional FOXA2-deficient EC cell line, MFE-319 (Supplemental Figure 1; supplemental material available online with this article; https://doi.org/10.1172/JCI157574DS1). Furthermore, scratch assays showed a significant delay in wound closure after FOXA2 reexpression (Figure 2, D and E). To further explore this apparent FOXA2-mediated cell growth suppression, cell cycle analyses were performed. FOXA2 inhibited cell cycle progression with an increase in cells in G0/G1 (ISK-*FOXA2*, 55.3% vs. ISK-EV, 39.8%) and a concomitant decrease of cells in S (ISK-*FOXA2*, 25.4% vs. ISK-EV, 31.9%) and G2/M (ISK-*FOXA2*, 19.3% vs. ISK-EV, 28.7%) phases (Figure 2F). In addition to these in vitro experiments, we analyzed the impact of FOXA2 reexpression in ISK cells through s.c. xenografts in immunocompromised mice. The FOXA2-expressing xenografts formed smaller tumors, grew more slowly (ISK-*FOXA2*, 144 ± 36 mm^3^ vs. ISK-EV, 652 ± 114 mm^3^), and weighed significantly less (ISK-*FOXA2*, 0.28 ± 0.05 g vs. ISK-EV, 1.33 ± 0.28 g) after 35 days (Figure 2, G–I).

To begin dissecting the biological basis of these FOXA2-induced effects, we then performed transcriptomic profiling of actively growing cells by RNA-Seq. FOXA2 reexpression exerted a profound effect on overall gene expression with 738 differentially expressed transcripts showing a more than 2-fold change at an arbitrary *P* value cutoff of less than 0.0001 with FDR less than 0.005 (Supplemental Table 1). *FOXA2* was the most highly upregulated gene (*P* < 10^–77^), serving as a positive control helping to validate the experiment (Supplemental Table 1). Intriguingly, we observed that *FOXA2* reexpression was associated with a concomitant upregulation of estrogen receptor (*ESR1*) and progesterone receptor (*PGR*) transcript abundance (Supplemental Table 1, *P* < 10^–100^ for either gene). This is analogous to the prior observation that FOXA1 binds to the androgen receptor enhancer and regulates its expression in prostate cancer cells (40). Gene Ontology (GO) analysis of the differentially expressed genes revealed a profound enrichment of GO categories relating to adhesion (see Figure 2J for selected categories, Supplemental Figure 2 for all of the GO categories, and Supplemental Table 2 for the list of genes in each category). These results are consistent with prior studies implicating FOXA2 in development as a driver of endoderm formation and specifically in the suppression of EMT (14), supporting the notion that FOXA2 is a classic oncodevelopmental factor with shared commonalities in its roles in morphogenesis and carcinogenesis. The impact of FOXA2 on cell cycle progression is likely explained at least in part by the observed downregulation of *MYC*, a potent driver of cell cycle progression (see also below) (Supplemental Table 1, *P* < 10^–97^; ref. 41). Next, we sought to validate the RNA-Seq results. Differential gene expression for several of the upregulated cell adhesion genes (Supplemental Figure 3A) was confirmed by qRT-PCR (Figure 2K). Cell adhesion assays of the ISK with or without *FOXA2* cells on surfaces coated with different extracellular matrix/adhesion factors showed significant enhancement of cell adhesion to multiple factors (Figure 2, L and M), confirming that FOXA2 is a potent general regulator of cell adhesion.

### FOXA2 knockdown in a FOXA2-expressing EC cell line promotes invasion and migration phenotypes through CDH1.

**Figure 3:**
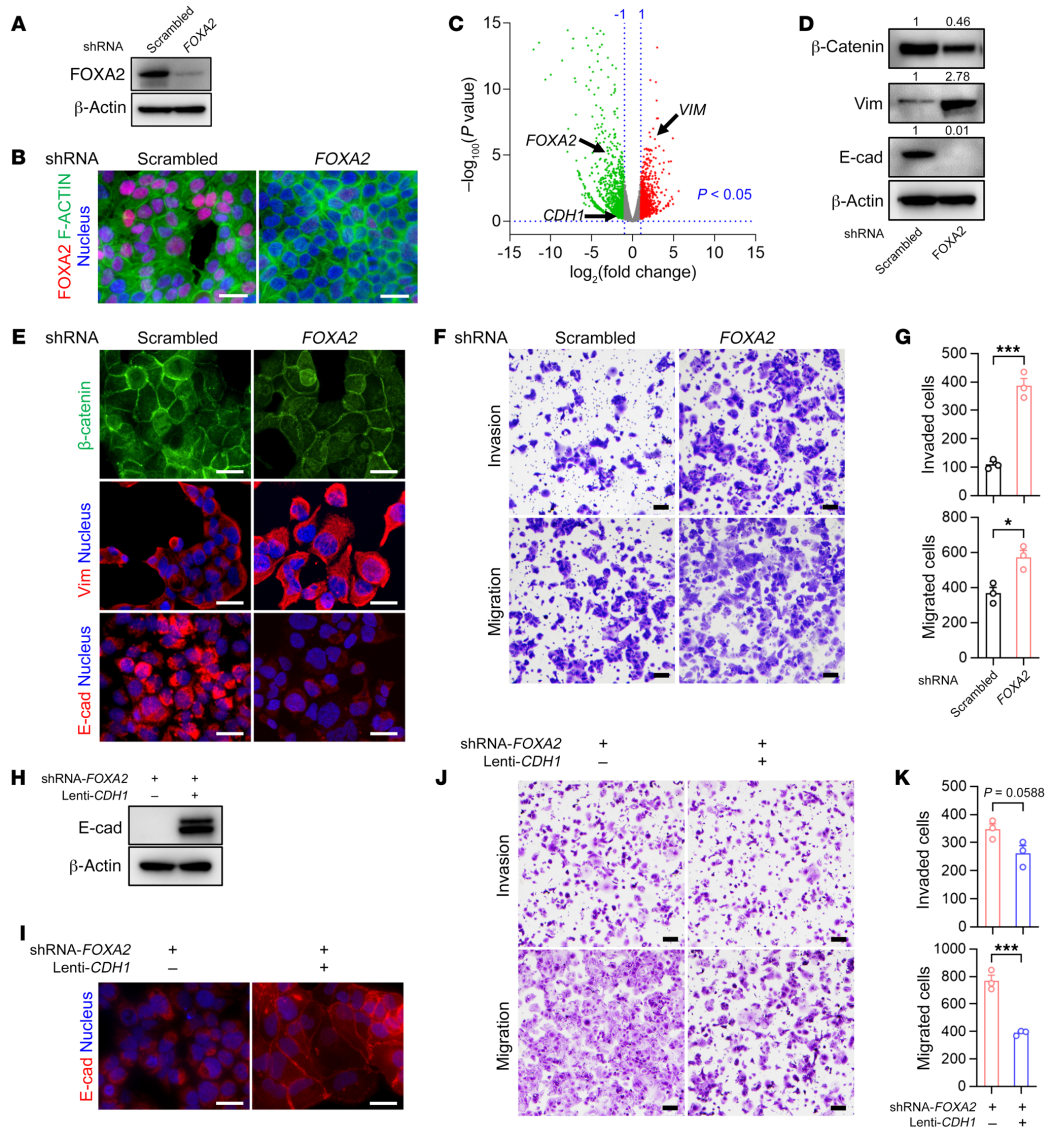
FOXA2 knockdown promotes EC cell migration and invasion. (**A**) Western blot shows effective lentiviral shRNA-mediated knockdown of *FOXA2* in HEC-1-B cells (one of the EC lines with high endogenous FOXA2 expression, Figure 1C). (**B**) Immunostaining of FOXA2 (red) with actin control (green) showing expected reduction of nuclear FOXA2 signal in *FOXA2*^KD^ HEC-1-B cells. Nuclei were stained with DAPI (blue). Scale bars: 50 μm. (**C**) RNA expression analysis, volcano plot showing differentially expressed significant genes (*P* <0.05) in *FOXA2*^KD^ HEC-1-B cells compared with empty vector control (1713 upregulated genes [red, fold change ≥ 2] and 2439 downregulated genes [green, fold change ≤ –2]). Dotted vertical lines represent log_2_ (fold change) threshold of ±1 and dotted horizontal line represents *P* value threshold of 0.05. Selected genes are shown. (**D**) Protein expression by Western blot in control and *FOXA2*^KD^ HEC-1-B cells. (**E**) Indirect immunofluorescence in control and *FOXA2*^KD^ HEC-1-B cells; representative images. Nuclei were stained with DAPI (blue). Scale bars: 50 μm. (**F**) Cell migration and invasion assays for control and *FOXA2*^KD^ HEC-1-B cells. Cells that migrated/invaded from the upper chamber of a Transwell to its lower chamber without (migration) or with a growth factor gradient (invasion) are visualized by crystal violet staining; representative images. Scale bars: 200 μm. (**G**) Quantitative analysis of migrated or invaded control and *FOXA2*^KD^ HEC-1-B cells (*n =* 3). Data shown as mean ± SEM; *P < 0.05; ***P < 0.001, 2-tailed *t* test. (**H**) Western blot documenting lentivirus-mediated enforced *CDH1* (E-cadherin) expression in *FOXA2*^KD^ HEC-1-B cells. (**I**) E-cadherin expression (red) by immunofluorescence in *FOXA2*^KD^ HEC-1-B cells with *CDH1* enforced expression. Nuclei were stained with DAPI (blue). Scale bars: 50 μm. (**J**) Cell invasion and migration assays of *FOXA2*^KD^ HEC-1-B cells with or without *CDH1* reconstitution. Scale bars: 200 μm. (**K**) Quantitative analysis of migrated and invaded *FOXA2*^KD^ HEC-1-B cells with or without *CDH1* reconstitution (*n =* 3). Data shown as mean ± SEM; ***P < 0.001, 2-tailed *t* test.

The observed loss of FOXA2 expression in poorly differentiated endometrial carcinomas and our FOXA2 reexpression studies (which revealed significant roles in cancer cell growth and promotion of cell adhesion to extracellular matrix factors) provoked the question of whether FOXA2 plays a role in EMT. To address this question, we performed *FOXA2* knockdown (KD) in HEC-1-B cells with a *FOXA2*-specific shRNA and with a nontargeting (scrambled) shRNA serving as negative control. *FOXA2*^KD^ HEC-1-B cells were subjected to antibiotic selection and robust KD was confirmed by Western blotting and immunofluorescence (Figure 3, A and B). Next, we performed transcriptional profiling of these 2 isogenic cell lines by RNA-Seq. Comparative profiling revealed 593 differentially expressed genes (>2-fold change and FDR < 0.01) in *FOXA2*^KD^ HEC-1-B cells compared with HEC-1-B cells (Figure 3C and Supplemental Table 3). Several EMT markers were identified in the differentially expressed gene list, including vimentin, E-cadherin, and β-catenin (Supplemental Figure 3B), and we confirmed aberrant expression of E-cadherin and vimentin by Western blot and immunofluorescence of *FOXA2*^KD^ HEC-1-B cells. Specifically, we found increased expression of the mesenchymal marker, vimentin, and concordant decrease in expression of the epithelial markers E-cadherin and β-catenin. These results demonstrated that *FOXA2* KD induced an EMT-like phenotype, indicating that one of FOXA2’s normal functions is the suppression of EMT in endometrial epithelium (Figure 3, D and E). To further explore the significance of this apparent EMT phenotype in *FOXA2*^KD^ HEC-1-B cells, we performed Transwell migration and invasion assays and found *FOXA2* KD significantly induced cell migration (*FOXA2*^KD^ HEC-1-B, 573 ± 38.7 vs. HEC-1-B, 367 ± 33.2) and invasion (*FOXA2*^KD^ HEC-1-B, 386 ± 26.2 vs. HEC-1-B, 110 ± 9.6) (Figure 3, F and G).

Previous studies demonstrated that FOXA1/2 proteins suppress EMT by activating E-cadherin expression in pancreatic cancer cells (42). Thus, in a *FOXA2-*KD EC cell line, we asked whether enforced E-cadherin expression would have any effect on EMT phenotypes. We transduced *FOXA2*^KD^ HEC-1-B cells with a lentivirus constitutively expressing a full-length cDNA for *CDH1*, which encodes E-cadherin. After antibiotic selection, Western blotting and immunofluorescence confirmed stable reexpression of E-cadherin (Figure 3H) with the expected cell membrane localization (Figure 3I). Next, to determine whether enforced E-cadherin expression had any effect on the cell migration and invasion phenotypes observed in the *FOXA2*^KD^ HEC-1-B cells, we carried out Transwell migration and invasion assays on these cells and ascertained that *CDH1* expression significantly suppressed cell migration (*FOXA2*^KD^/Lenti-*CDH1* HEC-1-B, 389 ± 10.7 vs. *FOXA2*^KD^ HEC-1-B, 769 ± 40.8) and to some extent cell invasion (*FOXA2*^KD^/Lenti-*CDH1* HEC-1-B, 262 ± 26.1 vs. *FOXA2*^KD^ HEC-1-B, 347 ± 19.2) (Figure 3, J and K). Together, these results confirmed that *FOXA2* suppressed EMT in EC cells and that these effects were mediated in part by E-cadherin.

### Foxa2/Pten mouse model establishes in vivo tumor suppressor functions of Foxa2 and potent synergism with Pten.

**Figure 4:**
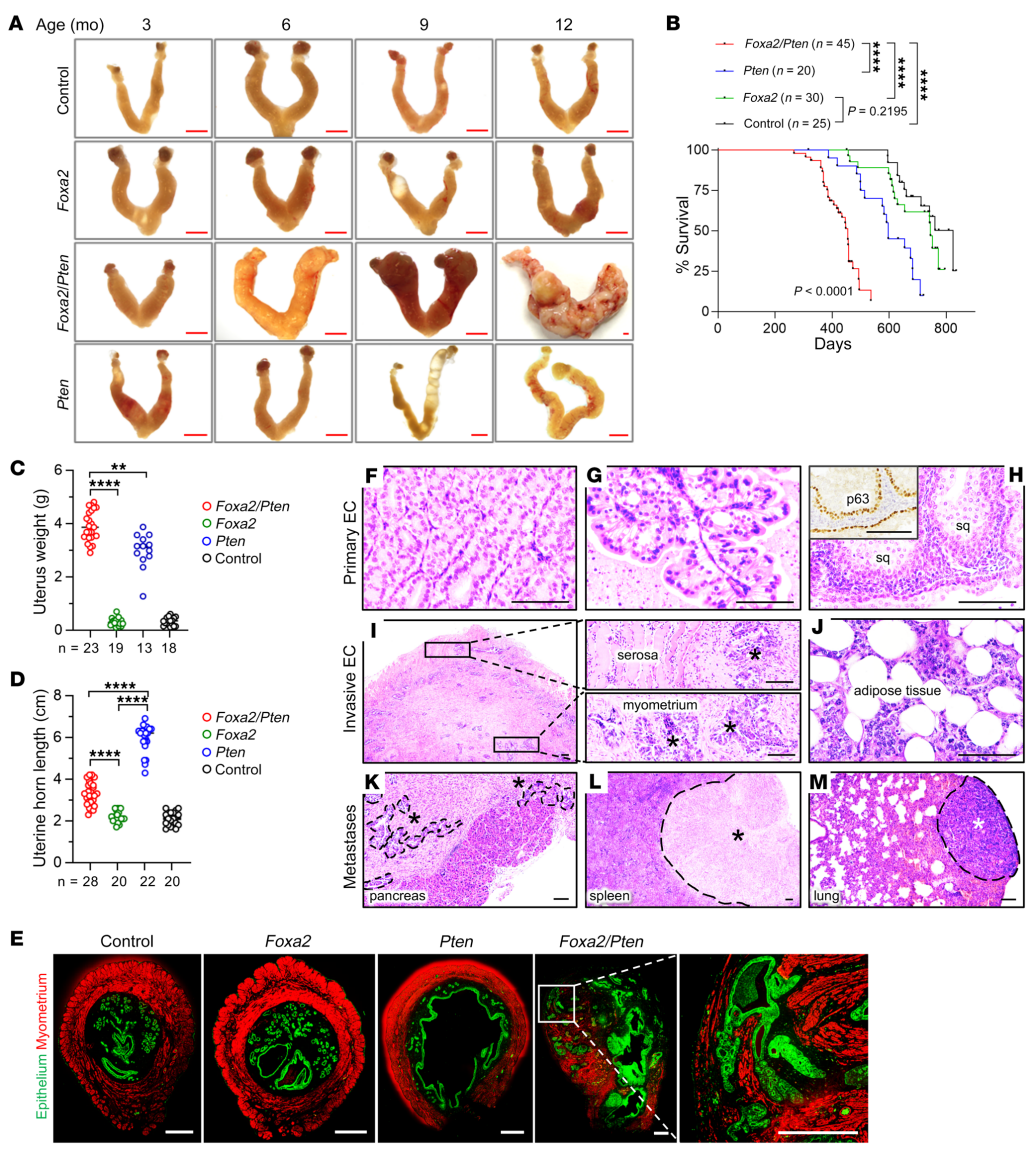
*Foxa2* functions as a tumor suppressor that cooperates with *Pten* in an in vivo conditional KO mouse model. (**A**) Gross images of uteri from control, *BAC-Sprr2f-Cre Foxa2^fl/fl^* (*Foxa2*), *Pten^fl/fl^* (*Pten*), or *Foxa2^fl/fl^Pten^fl/fl^* (*Foxa2*/*Pten*) mice at 3 to 12 months of age. Scale bar: 1 mm. (**B**) Survival analysis of *Foxa2*/*Pten* (*n =* 45), *Pten* (*n =* 20), and *Foxa2* (*n =* 30), and littermate control (*n =* 25) mice. ****P < 0.0001, log-rank test. (**C**) Uterine weights of *Foxa2*/*Pten* (*n =* 23), *Foxa2* (*n =* 19), *Pten* (*n =* 13), and littermate control (*n =* 18) mice at necropsy. **P < 0.01; ****P < 0.0001, 1-way ANOVA, Tukey’s multiple-comparison test. (**D**) Uterine horn lengths of *Foxa2*/*Pten* (*n =* 28), *Foxa2* (*n =* 20), *Pten* (*n =* 22), and littermate control (*n =* 20) mice. ****P <0.0001, 1-way ANOVA, Tukey’s multiple-comparison test. (**E**) Control, *Foxa2*, *Pten*, and *Foxa2*/*Pten* uteri (transverse sections) at 9 months of age immunostained with pan-cytokeratin (green) to label epithelial cells and αSMA (red) to label myometrium. Representative confocal tile-scan images are shown. Scale bars: 500 μm. (**F**–**M**) H&E-stained sections of *Foxa2*/*Pten* mouse cancers. (**F**) Well-differentiated adenocarcinoma. (**G**) Well-differentiated adenocarcinoma with papillary architecture. (**H**) Well-differentiated adenocarcinoma with squamous differentiation; inset shows p63 immunostain. sq, squamous differentiation. (**I**) Full-thickness myometrial invasion with higher magnifications of insets. (**J**) Infiltration into adjacent adipose tissue. (**K**–**M**) Abdominal and distant metastases marked by dashed black lines. Scale bars: 100 μm.

We then sought to explore the biological functions of *Foxa2* as an EC driver in an in vivo genetically engineered mouse model. First, we conditionally inactivated *Foxa2* with our endometrium-specific driver *BAC-Sprr2f-Cre*, which is estrogen-dependent and becomes active after the onset of sexual maturity at approximately 5 weeks of age (43, 44). *BAC-Sprr2f-Cre* thus would not interfere with normal prenatal or postnatal *Foxa2*-dependent uterine development or gland formation. *BAC-Sprr2f-Cre* was bred to mice harboring a floxed *Foxa2^fl^* allele, where *LoxP* sites flank exon 3. This exon encodes almost the entire protein, including the DNA binding domain, thus resulting in a definitive null allele following Cre-mediated recombination (45). *BAC-Sprr2f-Cre* *Foxa2^fl/fl^* females (abbreviated *Foxa2*) did not develop ECs up to 1 year of age, demonstrating that *Foxa2* inactivation alone was insufficient to drive ECs (Figure 4A) and suggesting that other cooperating genetic events may be necessary. This would also be consistent with the fact that most human ECs with *FOXA2* mutations harbor multiple oncogenic mutations. Several observations pointed to *Pten* inactivation as a particularly relevant cooperating genetic event. First, the PI3K pathway is frequently dysregulated in EC, and *PTEN*, a potent inhibitor of PI3K signaling, is the most frequently mutated gene in EC (46). Analyzing the Uterine Corpus EC TCGA data through cBioPortal (25), almost all *FOXA2* mutant cases also harbored mutations in the canonical PI3K pathway genes *PIK3CA*, *PIK3R1*, or *PTEN*, with *PTEN* being the most common. This cooperativity appeared to extend to cancers in general given that examination of all TCGA data sets revealed statistically significant co-occurrence of *FOXA2* and *PTEN* mutations (log_2_ odds ratio 1.30, *P* < 0.001).

Double-mutant *BAC-Sprr2f-Cre* *Foxa2^fl/fl^* *Pten^fl/fl^* mice (abbreviated *Foxa2*/*Pten*) were established for longitudinal studies and survival analysis along with single-gene KO cohorts. In contrast to *Foxa2*, *Foxa2*/*Pten* mice developed aggressive bulky uterine cancers by 1 year of age, resulting in greatly accelerated mortality (*P* < 0.0001, *Foxa2*/*Pten* vs. either single KO) (Figure 4, A and B). All *Foxa2*/*Pten* mice were confirmed at necropsy to have died of invasive EC. Survival analysis by log-rank test showed statistically significant differences in survival curves among the 3 groups (*P* < 0.01 for each of the pairwise comparisons). The median survival of *Foxa2*/*Pten* mice was significantly decreased compared with littermate controls or single KO mice (*Foxa2*/*Pten*, 455 days vs. *Pten*, 597 days vs. *Foxa2*, 745 days; *P* < 0.0001 per log-rank test) (Figure 4B). Uterine weights and histology also confirmed striking cooperativity between the 2 tumor suppressors relative to the single KO mice. *Foxa2* mice exhibited normal uterine weights, and while *Pten* uteri had increased weights, this was due to significantly longer uterine horns and not invasive cancers (Figure 4, C and D). The significance of the striking uterine length phenotype associated with *Pten* (5.9 ± 0.1 cm vs. controls, 2.1 ± 0.1 cm, *P* < 0.0001) was not further explored, but likely relates to the known role of the PI3K pathway in controlling Müllerian duct length (47). This *Pten*-mediated increase in uterine horn length was largely suppressed by concomitant inactivation of *Foxa2* in *Foxa2*/*Pten* uteri (*Pten*, 5.9 ± 0.1 cm vs. *Foxa2*; *Pten,* 3.3 ± 0.1 cm, *P* < 0.0001), although *Foxa2* alone uteri were indistinguishable from WT controls with respect to histology or length (Figure 4D). Immunofluorescence for cytokeratin and smooth muscle actin (to highlight epithelium and myometrium, respectively) confirmed full-thickness myometrial invasion only in the *Foxa2*/*Pten* uteri and not in the single KOs (Figure 4E).

Histologically, the primary ECs were characterized by well-formed malignant glands with occasional tumors exhibiting squamous differentiation confirmed by p63 IHC (Figure 4H). There were no obvious sarcomatous elements in any of the tumors. High-grade nuclear atypia (consistent with severe aneuploidy) was not observed, as in most prior mouse models (48–51). Thus, histologically, the *Foxa2*/*Pten* tumors resembled well-differentiated (i.e., grade 1) human endometrioid adenocarcinomas (Figure 4, F and G). Invasive cancers exhibited transmural invasion (Figure 4I) with extension into surrounding adipose tissue (Figure 4J), while metastases were found throughout the abdomen, including the pancreas (Figure 4K), liver, colon, and spleen (Figure 4L) (16/33 mice, 48%), with occasional mice harboring definitive hematogenous metastases to the lung (Figure 4M; 3/33, 9%). Loss of both Foxa2 and Pten in this model was confirmed by IHC. By only 2 months of age, *Foxa2*/*Pten-*deficient cells colonized the entire endometrium (Supplemental Figure 4). Since *BAC-Sprr2f-Cre* leads to subtotal mosaic Cre-mediated recombination with only 50% efficiency even in aged mice (43), this result further confirmed a potent growth advantage and outgrowth of the *Foxa2*/*Pten* mutant cells.

Initially, prior to the appearance of neoplasms, *Foxa2*/*Pten*-deficient but histologically normal glands retained ERα and PR, whereas invasive cancers in animals at 12 months of age showed ERα/PR loss (Supplemental Figure 4), similar to the loss of ERα/PR in human ECs (Figure 1, E and G). These results indicate that FOXA2 has an indirect (i.e., presumably not directly causal) impact on ERα/PR function in the endometrium that may be related to other more general aspects of EC progression. We then explored the possibility that *Tp53* mutations occur in the tumors by p53 IHC (a sensitive and specific surrogate of mutations) at necropsy in *n =* 23 mice from the survival analysis. None of the tumors exhibited p53 mutant (overexpressing) clones, which provides an argument that, unlike some other mouse models and human cancer types, p53 mutation is not essential for *Foxa2*-driven tumors (43, 44, 48, 51, 52). Taken together, these results demonstrated that *Foxa2* was a potent tumor suppressor where functional inactivation cooperated with *Pten* loss. Furthermore, the results validated this animal model for further investigations of *FOXA2* as an endometrial tumor suppressor.

### Functional studies using Foxa2/Pten primary tumor–derived organoids and cell lines show the roles of Foxa2 in suppressing cell growth and EMT phenotypes.

**Figure 5:**
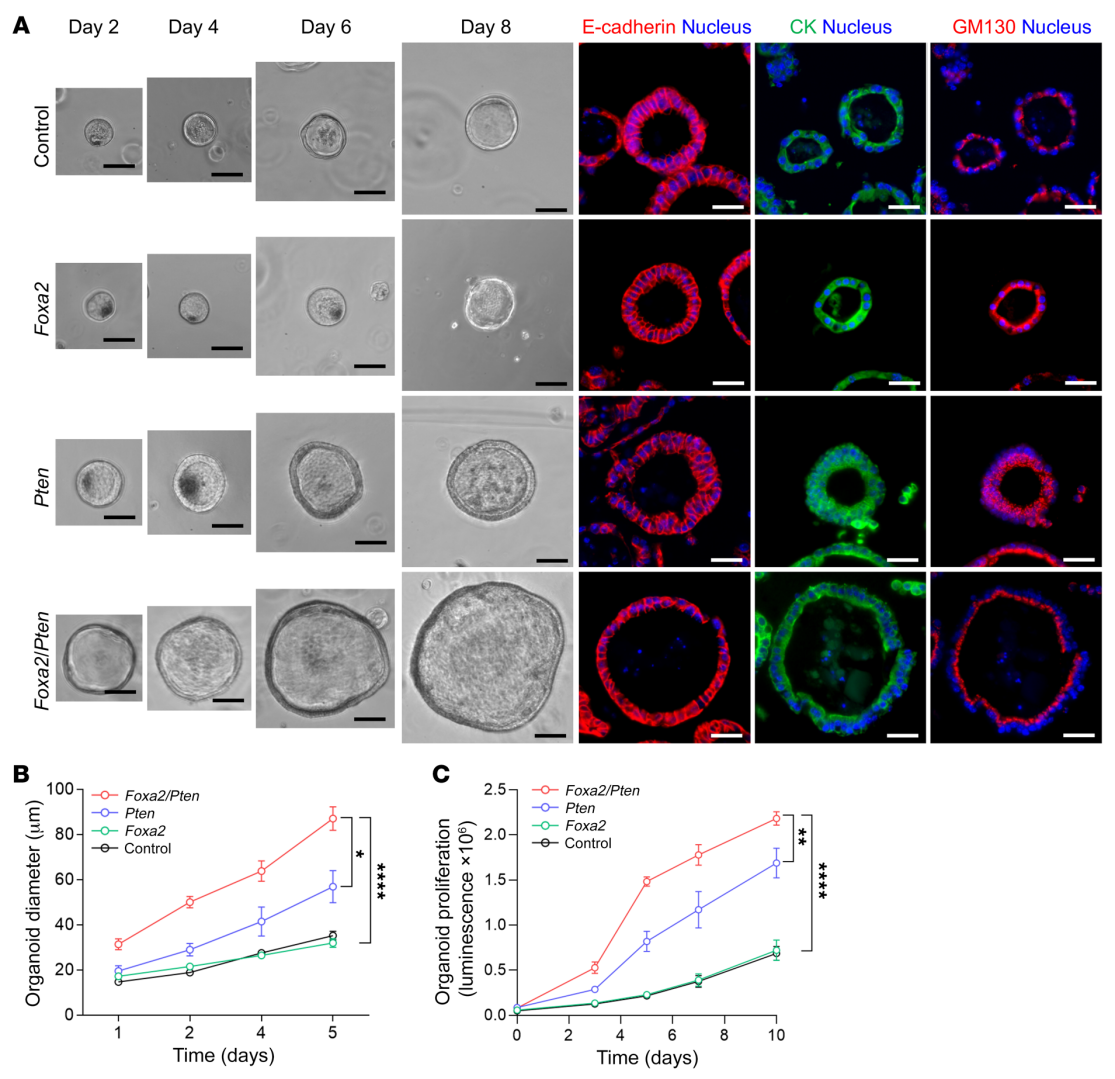
Synergism between *Foxa2* and *Pten* in 3D organoid growth. (**A**) Representative phase contrast images of control, *Foxa2*, *Pten*, and *Foxa2/Pten* mouse endometrial epithelial organoids at day 2 to day 8. Right panel shows E-cadherin, cytokeratin (CK), and GM130 expression on day 8 organoids by indirect immunofluorescence. Nuclei were stained with DAPI (blue). Scale bars: 50 μm. (**B** and **C**) Comparison of diameter and proliferation among organoids of differing genotypes (*n =* 3). Data shown as mean ± SEM; *P < 0.05; **P < 0.01; ****P < 0.0001, 2-tailed *t* test.

We generated endometrial organoids from *Foxa2*, *Pten*, and *Foxa2*/*Pten* mouse uteri at 2 months of age (Figure 5), by which time *Foxa2* and *Pten* are completely absent in endometrial glands. Generation of organoids well before the onset of neoplasia permits assessment of phenotypes without the confounding effects of additional alterations that may occur during malignant progression. *Foxa2* and *Pten* organoids were spherical with well-developed lumina. These organoids maintained normal epithelial cell polarity per GM130 (which labels the apical portion of the cell), E-cadherin, and cytokeratin (Figure 5A). *Pten* organoids grew faster and to a larger size than *Foxa2* organoids, whereas *Foxa2*/*Pten* organoids grew more rapidly and to an even larger size than *Pten* organoids (Figure 5, B and C). These results confirmed genetic cooperation among *Pten* and *Foxa2* and showed that *Foxa2* inactivation further potentiated *Pten*-associated growth phenotypes.

**Figure 6:**
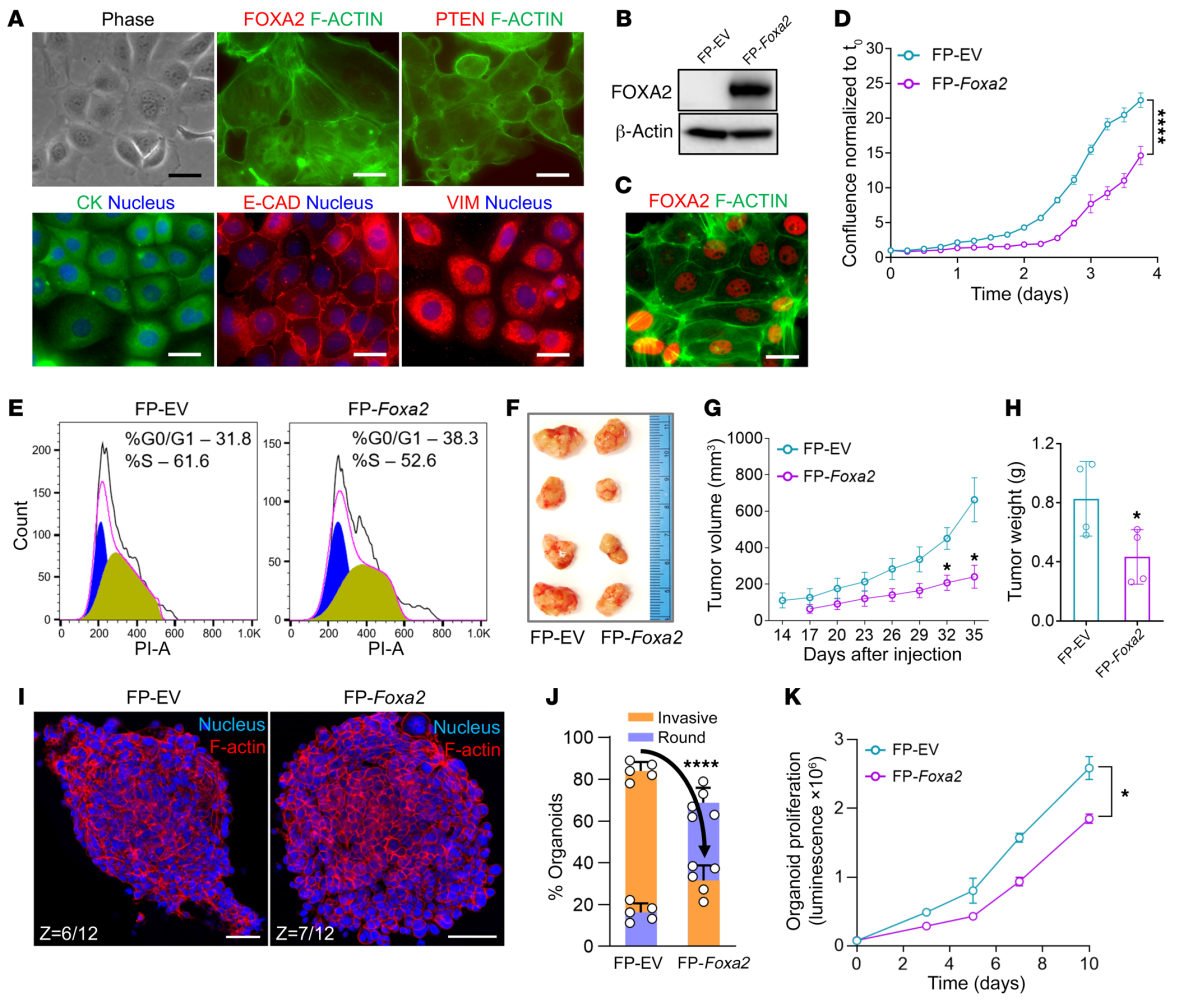
*Foxa2* reconstitution in FP cells suppresses cell growth and tumor phenotypes. (**A**) Brightfield and indirect immunofluorescence images of FP cell line derived from *Foxa2*/*Pten* mouse uterine tumor. Scale bars: 50 μm. (**B**) Western blot shows enforced *Foxa2* expression after lentiviral transduction in FP cells (FP-*Foxa2*). For controls, transduction was performed with empty vector (FP-EV). (**C**) Immunofluorescence of FOXA2 (red) validates nuclear localization in the FP-*Foxa2* cells. Scale bar: 50 μm. (**D**) Growth comparison of FP-EV and FP-*Foxa2* cells by real-time live cell imaging (*n =* 3). Data shown as mean ± SEM; ****P < 0.0001, 2-tailed *t* test. (**E**) Comparison of cell cycle analysis in FP-EV and FP-*Foxa2* cells by flow cytometry (*n =* 3). The peaks in blue and yellow show percentage of cells in G0/G1 and S phase. (**F**) Gross images of xenograft tumors after s.c. injection of 1 million FP-EV and FP-*Foxa2* cells in the left and right flanks of NOD *scid* gamma female mice (*n =* 4). Tumors were harvested 35 days after cell injection. (**G**) Growth curves of FP-EV and FP-*Foxa2* xenografts per caliper measurements (*n =* 4). Data represent mean ± SEM; *P < 0.05, 2-tailed *t* test. (**H**) Endpoint xenograft tumor weights at day 35 (*n =* 4, same tumors shown in **F**). Data represent mean ± SEM; *P < 0.05, 2-tailed *t* test. (**I**) Immunostaining of actin filaments (red) in FP-EV and FP-*Foxa2* organoids counterstained with DAPI (blue). Representative midsections of *Z*-stack images are shown. Scale bars: 50 μm. (**J**) Quantitative analysis of invasive or round-shaped FP-EV and FP-*Foxa2* organoids (*n =* 5). Data shown as mean ± SEM; ****P < 0.0001, 2-tailed *t* test. (**K**) Comparison of FP-EV and FP-*Foxa2* organoid proliferation (*n =* 4). Data shown as mean ± SEM; *P < 0.05, 2-tailed *t* test.

Then, we generated the “FP” cell line from a *Foxa2*/*Pten* malignant EC harvested at 1 year of age. When grown on standard culture conditions on plastic, FP cells maintained characteristic cobblestone epithelial cell morphology and expressed cytokeratin and E-cadherin but also vimentin, suggesting some degree of EMT (Figure 6A). Lentiviral transduction of FP cells with a full-length *Foxa2* cDNA (FP-*Foxa2*) resulted in stable reexpression and nuclear localization of Foxa2 protein (Figure 6, B and C). *Foxa2* reexpression resulted in significantly decreased cell growth (Figure 6D) associated with increased numbers of cells in G0/G1 phase (FP-*Foxa2*, 38.3% vs. FP-EV, 31.8%) and decreased cells in S phase (FP-*Foxa2*, 52.6% vs. FP-EV, 61.6%) (Figure 6E). As s.c. allografts, FP-*Foxa2* tumors grew more slowly (FP-*Foxa2*, 240 ± 62.9 mm^3^ vs. FP-EV, 662 ± 121.1 mm^3^) and to lower final weights at 35 days (FP-*Foxa2*, 0.43 ± 0.09 g vs. FP-EV, 0.83 ± 0.13 g) (Figure 6, F–H). These results are consistent with the results obtained with the human ISK EC cell line (Figure 2, C–I).

Next, we generated organoids from FP-EV cells. FP-EV organoids grown under standard conditions in Matrigel (53, 54) exhibited highly irregular shapes with pseudopodia-like extensions (Figure 6I and Supplemental Figure 5). After *Foxa2* reexpression, FP cells reverted to completely spherical shapes with well-defined boundaries as evidenced by analyses of single-plane images, *Z*-stack images, and videos (Figure 6I, Supplemental Figure 5, and Supplemental Video 1). Quantitatively, 84% ± 1.9% of FP-EV organoids displayed invasive pseudopodia, whereas only 31.2% ± 3.2% (*P* < 0.0001) of FP-*Foxa2* organoids did so (Figure 6J). Comparison of long-term growth of FP-EV and FP-*Foxa2* organoids for 10 days showed that *Foxa2* expression also suppressed cell proliferation (Figure 6K). Taken together, these studies of FP-EV organoids and cell lines extend and further validate our previous data that Foxa2 controls cell proliferation and also regulates pro-invasive EMT phenotypes.

### Transcriptional profiling of mouse-derived Foxa2/Pten EC cell line +/– Foxa2 reexpression shows Foxa2 suppresses EMT during EC progression.

To further explore specific biological roles of *Foxa2* in the murine system, transcriptional profiling by RNA-Seq was performed on FP-EV versus FP-*Foxa2* cells. Foxa2 reexpression resulted in a profound transcriptional reprogramming with differential expression of 806 genes (373 up- and 433 downregulated) per stringent criteria, including more than 2-fold change and *P* less than 0.001, FDR less than 0.005 (Supplemental Table 4). Some of the differentially expressed genes included cancer progression factors, such as *Tff1* (trefoil factor 1), *Anxa10* (annexin a10), *Gnai1* (G protein subunit alpha i1), *Areg* (amphiregulin), and *Mmp7* (matrix metalloproteinase 7) (Supplemental Figure 6A). *Foxa2* induced expression of several developmental factors, including *Fgf13* (fibroblast growth factor 13), *Ngf* (nerve growth factor), and *Ctse* (cathepsin E) (Supplemental Figure 6A). GO analysis identified several categories aligned with Foxa2’s participation in tissue and developmental processes, including tube and structure morphogenesis, further supporting Foxa2’s status as an oncodevelopmental factor with shared functions in adenogenesis (refs. 18–20 and 55; Supplemental Figure 6B). Additionally, the upregulated GO categories included regulation of cell adhesion and negative regulation of cell proliferation (Supplemental Figure 6B). In contrast, downregulated GO categories included regulation of cell migration and EMT (Supplemental Figure 6C). To further validate misexpression of potential factors in EMT-related GO categories, we analyzed the expression pattern of several EMT-related factors by qRT-PCR and Western blot analysis. In FP-*Foxa2* cells, there was significant downregulation of N-cadherin (*Cdh2*), vimentin (*Vim*), Slug (*Snail2*), and *Zeb2*, which were also partly confirmed by Western blot (Supplemental Figure 6, D and E). However, expressions of other EMT regulators, such as β-catenin (*Ctnnb1*), E-cadherin (*Cdh1*), Snail (*Snail1*), and *Zeb1*, were not noticeably altered (Supplemental Figure 6D). Additionally, qRT-PCR confirmed significant upregulation of cell adhesion genes, including *Agr2* and *Cntn1* in FP*-Foxa2* cells (Supplemental Figure 6F). These results are concordant with our earlier results in human EC cell lines where *FOXA2* reexpression and KD promoted cell adhesion and EMT-related phenotypes.

### FOXA2 regulates the EC transcriptome by shaping the enhancer landscape.

Our results provide compelling evidence that FOXA2 is a tumor suppressor in EC. Enhancers are DNA elements that govern cell type–specific gene expression by recruiting transcription factors and regulating RNA polymerase activity. Enhancers can be located anywhere from inside gene bodies to noncoding regions hundreds of kilobase pairs from their target genes. As a pioneer transcription factor in this context, FOXA2 should bind to large numbers of genome-wide regulatory regions — such as enhancers — to control gene expression. We hypothesized that by regulating key transcriptional programs, FOXA2 has an essential role in establishing and maintaining the endometrial epithelial lineage. We next conducted integrative genomics and computational analysis to unravel such mechanistic links between FOXA2 and malignant transformation of the endometrium.

**Figure 7:**
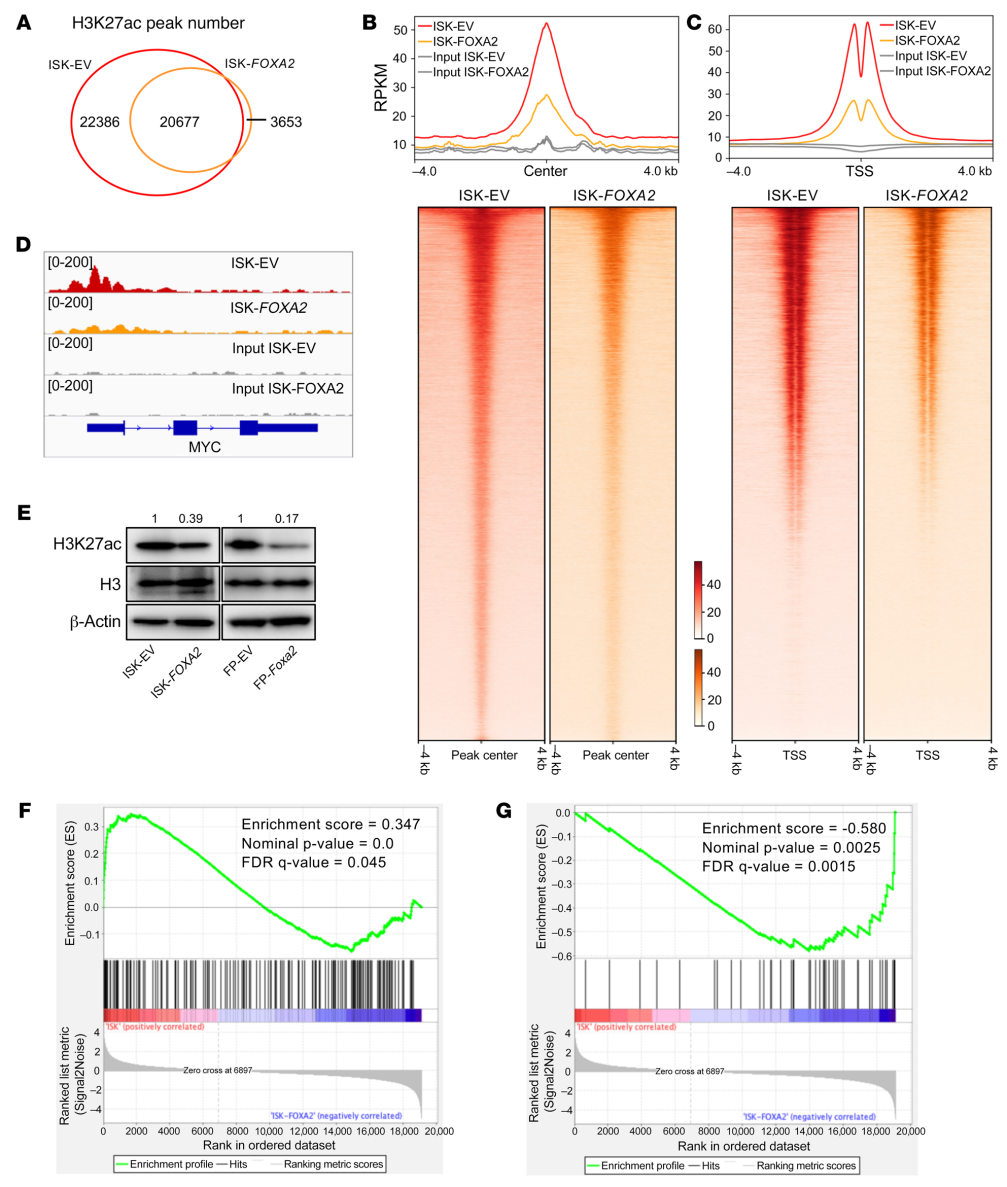
Transcriptional reprogramming by FOXA2. (**A**) Venn diagram representing H3K27ac peaks. (**B**) Average coverage plot and heatmap representation of ChIP-Seq signals ±4 kb around acetyl histone (H3K27ac) peaks. (**C**) Average coverage plot and heatmap representation of ChIP-Seq signals ±4 kb around transcriptional start sites. (**D**) Genome browser representation of H3K27ac peaks in the *MYC* gene. (**E**) Western blot analysis of H3 and H3K27ac protein expression in ISK-EV, ISK-*FOXA2*, FP-EV, and FP-*Foxa2* cells. (**F** and **G**) Gene set enrichment analysis (GSEA). Genes shaded with red in the heatmap are upregulated in parental ISK cells (in comparison to ISK-FOXA2). Genes shaded with blue in the heatmap are upregulated in ISK-FOXA2 (in comparison with parental ISK cells).

ISK cells do not express FOXA2 but do express the steroid hormone receptors ERα and PR. We thus sought to determine the effect of FOXA2 reexpression on the enhancer landscape of ISK cells. We conducted ChIP-Seq analysis of H3K27ac (acetylation of histone H3 at lysine 27), a mark of active enhancers and active promoters in parental ISK (ISK-EV) and FOXA2-expressing ISK cells (ISK-*FOXA2*). We identified approximately 43K H3K27ac peaks in parental ISK cells. Remarkably, upon FOXA2 reexpression, the H3K27ac signal was lost in about half of these peaks (~22K), indicating loss of enhancer activity or enhancer decommissioning (Figure 7, A and B). FOXA2 reexpression was also associated with the formation of new enhancers with the H3K27ac signal (*n =* 3653). The majority of decommissioned enhancers were in distal regulatory regions (*n =* 19,966) and only 2420 were promoter proximal enhancers (–500 bp to +250 bp of transcription start sites [TSS]). Likewise, the majority of new enhancers were in distal regulatory regions (*n =* 3555) and only 98 were promoter proximal enhancers. The common enhancers (*n =* 20,677) were equally represented by distal regulatory regions (*n =* 10,308) and promoter proximal regions (*n =* 10,369). These data indicate that FOXA2 reexpression contributed to massive enhancer reprogramming in ISK cells — particularly in the distal regulatory regions.

Next, we conducted focused analyses of TSS (Figure 7C). The signature bimodal peak is suggestive of nucleosome-free regions in TSS, a feature of expressed genes. FOXA2 reexpression was associated with an overall reduction in H3K27ac signal strength in the TSS. We next determined the relationship between changes in H3K27ac signal and gene expression changes. We identified 1622 genes with promoter regions overlapping with FOXA2-mediated decommissioned H3K27ac peaks (*n =* 22,386). Then, we took the top 10% of those genes with the highest H3K27ac enrichment in parental ISK cells (*n =* 162) and performed gene set enrichment analysis (GSEA; refs. 56, 57) using the RNA-Seq expression profiles of ISK-EV and ISK-*FOXA2* samples. These 162 genes were significantly enriched in the genes upregulated in parental ISK cells (in comparison to ISK-*FOXA2*) (Figure 7F). We identified 45 genes with promoter regions overlapping with the new enhancers commissioned by FOXA2 reexpression (*n =* 3653). GSEA indicated that these 45 genes were significantly enriched in the genes upregulated in ISK-*FOXA2* (in comparison to parental ISK cells) (Figure 7G). Taken together, these results showed that FOXA2 functions as a multitasking endometrial tumor suppressor by regulating the transcriptome through shaping the enhancer landscape.

We documented that the expression of several EC genes was dysregulated upon FOXA2 reexpression (Figure 2 and Supplemental Table 1). In particular, we were intrigued by FOXA2-mediated downregulation of *MYC*, a classic oncogene, an attractive candidate as a mediator for observed phenotypes in, e.g., cell proliferation. We therefore focused on *MYC* transcriptional regulation, the accompanying epigenetic changes in the *MYC* gene, and the concomitant effects on *MYC* target genes upon FOXA2 reexpression. FOXA2 reexpression was associated with loss of H3K27ac in the promoter, +1 nucleosome, and gene body of *MYC* (Figure 7D). Consistent with this observation, FOXA2 reexpression was associated with downregulation of MYC protein and various cyclin-dependent kinases regulated by MYC in both human (ISK-EV) and mouse EC cell lines (FP-EV) (Supplemental Figure 7A and ref. 58). Lentiviral transduction of full-length *Myc* cDNA in FP-*Foxa2* cells reversed the effects on cyclin-dependent kinases and resulted in increased cell proliferation (*P* < 0.0001) following *Myc* reexpression (Supplemental Figure 7, B and C). These results demonstrated that MYC is a direct target negatively regulated by FOXA2 activity through enhancer binding. Importantly, FOXA2 reexpression resulted in a reduction in the steady-state levels of H3K27ac (Figure 7E). We conducted additional studies to explore the generalizability of these observations with the mouse *Foxa2/Pten* EC cell line (FP) described above. Consistent with the data observed in ISK cells, Foxa2 reexpression via lentivirus (FP-*Foxa2*) was associated with a reduction in the steady-state levels of H3K27ac (Figure 7E). Overall, these results indicated that FOXA2 regulated enhancer activity in human and mouse EC cells.

## Discussion

Diverse observations in this study, made in humans and mice, combined with previous studies, designate *FOXA2* as a tumor suppressor where loss of function promotes endometrial carcinogenesis. First, we documented that FOXA2 protein was downregulated in primary human ECs in a grade-dependent manner and was lost in most grade 3 cancers, suggesting that there is strong selection for FOXA2 loss during EC progression. Second, we showed that in a panel of 13 independently derived EC cell lines, FOXA2 was undetectable in more than half the cell lines (7/13), consistent with a prior study (23). In another 4/13 of the cell lines, FOXA2 protein was detectable but at markedly decreased levels. Third, reexpression of WT FOXA2 in the well-differentiated ISK EC cell line led to pronounced suppression of growth– and other cancer–related phenotypes. Fourth, shRNA-mediated KD of *FOXA2* in the *FOXA2*-WT EC cell line HEC-1-B resulted in striking EMT-related phenotypes, including pro-invasive and pro-migration phenotypes via the suppression of E-cadherin. Fifth, and most compellingly, conditional ablation of the *Foxa2* gene in live animals via an endometrial epithelium–specific Cre driver led to the formation of highly invasive and lethal ECs. This striking cancer phenotype occurred only with simultaneous inactivation of *Pten* (which by itself resulted only in the formation of noninvasive/nonlethal hyperplasias), establishing potent synergism between *Foxa2* and this canonical endometrial tumor suppressor. Of note, almost the entire *Foxa2* open reading frame was deleted in this mouse model, resulting in complete loss of *Foxa2* function (45). This mouse model thus provides formal genetic proof that *Foxa2* functions as a tumor suppressor. Sixth, diverse analyses of cell lines and organoids derived from the mouse *Foxa2*/*Pten* primary ECs further supported a tumor suppressor role for *Foxa2* via the suppression of EMT-associated invasive and metastatic phenotypes.

These results are provocative in light of compelling recent data that for prostate cancer, *FOXA1* mutations are generally heterozygous and gain of function (genetically dominant, making *FOXA1* an oncogene) (26, 30). However, there are significant differences in the FOXA1 and FOXA2 amino acid sequences, which have only 51% identity in humans. Thus, despite their similarity and evidence for some functional redundancy, substantial differences in their function and regulation are likely. Concordantly, differences in *FOXA1* and *FOXA2* mutational spectra in cancer are striking. First, unlike *FOXA1*, *FOXA2* mutations are not highly clustered in the forkhead amino acid domain or elsewhere in the protein. Of 97 *FOXA2* point mutations, only 2 occurred more than twice, and both occurred only 3 times. Of the 97 mutations, 24 were truncating and were evenly distributed across the *FOXA2* open reading frame, without significant clustering in the C-terminus as occurs for *FOXA1* (25). Recurring structural rearrangements involving *FOXA2* have not been described (21). The dearth of recurring mutations in *FOXA2* is consistent with our finding that *FOXA2* is a tumor suppressor. However, more detailed genomic investigations into the range of mutations affecting the *FOXA2* locus in EC are warranted, and it is possible that novel mutation classes remain to be discovered.

There are also likely to be differences in FOXA1 and FOXA2 function mediated by fundamentally different steroid receptor biology in diverse organs. For example, prostate biology is largely driven by the androgen receptor, with FOXA1 directly binding to the androgen receptor and serving as its pioneer transcription factor. FOXA2 has been less studied than FOXA1, but FOXA2 also serves as a pioneer transcription factor for ERα in the endometrium (59), and our data directly support such a pioneer factor role for FOXA2 in the endometrium. There have been no direct studies of interactions between FOXA2 and PR in the endometrium, but ERα and PR-A/B have largely opposing actions. Such interplay and further complexities in the oscillating levels of estrogen and progesterone during each reproductive cycle in women may also underlie fundamental differences in the biological roles of FOXA1 versus FOXA2 in the prostate and endometrium or other tissues and contribute to differences in mutational spectra.

Inactivation of a single *FOXA2*/*Foxa2* allele (haploinsufficiency) can result in distinct phenotypes. *Foxa2^+/–^* heterozygous mice fed a high-fat diet developed increased adiposity as a result of decreased energy expenditure (60). In humans, heterozygous deletions or point mutations of *FOXA2* are associated with a genetic syndrome characterized by diverse organ defects, including pituitary abnormalities (61–63). Studies of human ECs have also raised the possibility that *FOXA2* haploinsufficiency contributes to carcinogenesis. For example, the majority of primary ECs with documented *FOXA2* mutations had only one identifiable mutation and did not exhibit apparent loss of heterozygosity, although some tumors did exhibit definitive biallelic mutations. Furthermore, in these cases, the mutations were in *trans*, suggesting that there was selection for biallelic loss (22). In practice, it can be difficult to definitively distinguish haploinsufficiency from spontaneous loss of the second allele due to a wide variety of genetic perturbations that can inactivate an allele, some of which may be undetectable by standard sequencing or loss-of-heterozygosity analysis. This question could be explored in the future in mouse models by, e.g., generation of a conditional *Pten*^+/+^ *Foxa2^+/–^* cohort. However, even if this genotype exhibited intermediate survival (between *Foxa2^+/+^* and *Foxa2^–/–^*), it might be difficult to distinguish between true haploinsufficiency and spontaneous loss of the second allele, which is common for many tumor suppressor loci in mouse models (e.g., *Pten*; refs. 64, 65).

Scheibner et al. investigated the role of Foxa2 during endoderm formation in mice using knockin/KO fluorescent protein reporters. In homozygous *Foxa2^Venus^* mice, there is a lack of definitive endoderm formation. Interestingly, the EMT transcription factor genes *Snail* and *Zeb1/2* and the EMT marker genes *Vimentin*, E-cadherin (*Cdh1*), and N-cadherin (*Cdh2*) are upregulated in *Foxa2* mutant endoderm precursor cells. In these cells, *Foxa2* suppresses Snail to prevent E-cadherin downregulation and EMT. Taken together, these results show that Foxa2 acts as an “epithelial gatekeeper and EMT suppressor” to prevent endoderm progenitors from undergoing EMT (14). In line with these results, we found in an open-ended gene discovery effort with a mouse EC cell line that *Foxa2* KD resulted in aberrant expression of EMT markers, including *Vimentin*, *Zeb1/2*, and *Cdh1/2* (34). We also found evidence for a role of Foxa2 in EMT suppression in organoids derived from *Foxa2*/*Pten* primary mouse endometrial tumors; for example, Foxa2 reexpression caused decreased growth and striking loss of both cellular organization and pseudopodial extensions. These results establish links between the functions of FOXA2 in embryonic development and in cancer progression. FOXA2 has also been implicated as an EMT antagonist in pancreatic, colon, and lung cancers (42, 66–68).

The biological basis of the observed synergism between *Foxa2* and *Pten* is uncertain, but some inferences can be drawn. Mutational inactivation of *Pten* (or other PI3K pathway components) act principally (though not exclusively) as drivers of cellular proliferation and cell cycle progression through effectors such as Gsk3 and the Foxos (7, 43). Consistent with such a principal role of PI3K pathway misregulation in driving cell growth, *Pten* inactivation in the endometrial epithelium (including in mouse models) drives hyperplasia but not invasive cancers (43, 69). Our *Pten*-alone conditional KO controls confirmed this, as did our analyses of *Pten* endometrial organoids showing a marked propensity for accelerated growth. However, Pten loss is a relatively poor driver of EMT, as evidenced by a lack of invasive phenotypes in Pten-alone contexts in this study, including live mice, cell lines, and organoids. This is also well-documented in aging human endometrium, where definitive Pten loss occurs in normal or hyperplastic endometrium (i.e., very early in EC progression, well before the acquisition of invasive phenotypes) (24, 70, 71). In contrast, in our studies, Foxa2 inhibited cell cycle progression via Myc, albeit modestly, confirming that it is a multitasking tumor suppressor with diverse biological roles, but with a much more potent effect as an EMT suppressor, as evidenced by upregulation of EMT factors and the striking pro-invasive/pro-metastatic phenotypes observed in the live animal and human/mouse cell line and organoid systems. These observations lead us to propose that the synergism between Foxa2 and PI3K pathway activation in endometrial and other cancers is a consequence of their impact on distinct biological processes of cell cycle progression and EMT that are critical for the acquisition of invasive and metastatic phenotypes.

## Methods

### Mouse breeding.

Mice harboring the floxed *Foxa2* (*Foxa2^tm1Khk^*/J, stock 022620) and *Pten* (*Pten^tm1Hwu^*/J, stock 004597) alleles were obtained from the Jackson Laboratory. Homozygous conditional deletion in endometrial epithelium was conducted by breeding to *BAC-Sprr2f-Cre* (43, 44). This allele in a pure C57BL/6J background [B6(FVB)-Tg(Sprr2f-Cre)2Dcas/J] will be available from the Jackson Laboratory Repository (stock 037052). Mice were housed in a pathogen-free animal facility in individually ventilated cages and fed a standard chow diet ad libitum.

### Human tissue.

EC tissue microarray tissue sections (US Biomax, EMC1021) were used for IHC. Normal human endometrial and prostate tissue sections were obtained in an anonymized manner from FFPE tissue blocks from the UT Southwestern Tissue Resource, an IRB-approved institutional core facility.

### Data availability.

The ChIP-Seq and RNA-Seq data that support the findings of this study have been deposited in the NCBI’s Gene Expression Omnibus (GEO) and are accessible through GEO accession numbers GSE193165 and GSE197211.

### Statistics.

All statistical analyses were performed with GraphPad Prism (v.9.1.2). Data are presented as the mean ± SEM unless otherwise indicated. Statistical analysis was performed with Student’s *t* test (unpaired, 2-tailed). Comparisons among multiple groups were performed using 1-way ANOVA. FOXA2 protein expression levels were compared across groups using the Mann-Whitney *U* test. Pearson’s analysis was performed to determine the correlation between the groups. Differences between survival curves were estimated by Kaplan-Meier analysis and log-rank test. *P* less than 0.05 and FDR less than 0.05 were considered statistically significant.

### Study approval.

The UT Southwestern IACUC approved all the animal procedures and experiments.

See Supplemental Methods for additional details.

## Author contributions

SSS, RSM, and DHC conceived the study. SSS, SGR, YG, AA, AK, ICC, HDL, MA, and CX performed experiments and analyzed data. DHC, RSM, and SSS wrote the manuscript with input from all authors.

## Supplementary Material

Supplemental data

Supplemental tables 1-4

Supplemental video 1

## Version 1 06/15/2022

Electronic publication
