# Supplementary material for: FOXA2 suppresses endometrial carcinogenesis and epithelial-mesenchymal transition by regulating enhancer activity
Source: J Clin Invest. 2022 Jun 15;132(12):e157574. doi: 10.1172/JCI157574 (PMC9197528; doi:10.1172/JCI157574)
Supplement: Supplemental data [file jci-132-157574-s099.pdf]

Supplemental Information

FOXA2 suppresses endometrial carcinogenesis and epithelial-mesenchymal transition by regulating enhancer activity

Subhransu S. Sahoo[1], Susmita G. Ramanand[1], Yunpeng Gao[1], Ahmed Abbas[1], Ashwani Kumar[2], Ileana C. Cuevas[1], Hao-Dong Li[1], Mitzi Aguilar[1], Chao Xing[2,3,4], Ram S. Mani[1,5,6], Diego H. Castrillon[1,5,7]

[1]Department of Pathology

[2]Eugene McDermott Center for Human Growth and Development

[3]Department of Bioinformatics

[4]Department of Population and Data Sciences

[5]Harold C. Simmons Comprehensive Cancer Center

[6]Department of Urology

[7]Department of Obstetrics and Gynecology

UT Southwestern Medical Center, Dallas, TX 75390-9072, USA

Address correspondence to: Diego H. Castrillon, Department of Pathology, UT Southwestern Medical School, 6000 Harry Hines Boulevard, NB6.452, Dallas, TX 75390-9072 USA. Phone: 214-648-4032; E-mail: diego.castrillon@utsouthwestern.edu

Supplemental Methods

Endometrial cancer (EC) cell lines

a) Mouse EC cell line (FP) derivation and culture

A mouse EC cell line (FP) was established from a fresh endometrial tumor derived from a one-year-old *BAC-Sprr2f-Cre*; *Foxa2*$^{L/L}$, *Pten*$^{L/L}$ mouse. The tumor was washed with PBS, cut into 2-3 mm fragments with a sterile surgical scalpel and digested in Collagenase V/Dispase II enzymatic solution (0.4 mg/ml Collagenase V and 1.25 U/ml Dispase II: Sigma) in Advanced DMEM/F12 (Thermo Fisher Scientific) at 37^{0}C for 1 hr with intermittent shaking. Tissue digestion was stopped by adding Advanced DMEM/F12 containing 10% FBS (Sigma). The tumor fragments were triturated 15-20 times and centrifuged at 800 rpm for 5 min. The isolated cells were washed twice in Advanced DMEM/F12 and cultured in Advanced DMEM/F12 medium containing 10% FBS and 1% penicillin-streptomycin (Thermo Fisher Scientific) at 37^{0}C, 5% CO_2. The cells were passaged 3 times to obtain pure epithelial cells and the characteristic cobblestone epithelia cell morphology was confirmed by phase contrast microscopy.

b) Human EC cell lines

Human EC cell lines (Ishikawa, MFE-319, EN, AN3CA, HEC-1-A, HEC-1-B, EI, EJ, EFE-184, MFE-280, MFE-296, RL95-2, KLE) were cultured in standard media per ATCC recommendations supplemented with heat-inactivated FBS and antibiotics (50 U/mL penicillin and 50 mg/L streptomycin) in a humidified atmosphere at 37^{0}C containing 5% CO_2. A normal human endometrial epithelial cell line (HEuEC, Lifeline Cell Technology #FC-0078) was maintained in ReproLife reproductive medium (Lifeline Cell Technology #LL-0068). Cell lines were confirmed to be *Mycoplasma* negative with the Mycosensor PCR assay kit (Agilent Genomics #302108).

c) Lentiviral transduction

To re-express *FOXA2*, Ishikawa, MFE-319 and FP cells were transduced with pLenti-C-Myc-DDK vector only (ISK-EV, MFE-319-EV, FP-EV) or vector containing human and mouse *FOXA2* (ISK-

FOXA2, MFE-319-*FOXA2*, FP-*Foxa2*) ORF clone (OriGene) following manufacturer's instructions. Stable cell lines were obtained following puromycin selection. For *Myc* expression, mouse FP-*Foxa2* cells were transduced with pLenti-C-Myc-DDK vector containing mouse full length *Myc* (FP-*F*oxa2-*Myc)* gene (G&P Bioscience) following manufacturer's instructions. *FOXA2* was knocked down in HEC-1-B cell line through a 29-mer *FOXA2* specific shRNA (*FOXA2*^{KD} HEC-1-B) and through a 29-mer scrambled shRNA in pGFP-C-shLenti vector (OriGene) with puromycin selection. To enforce E-cadherin protein expression, *FOXA2*^{KD} HEC-1-B cells were transduced with pLenti-C-Myc-DDK vector only (*FOXA2*^{KD} HEC-1-B-EV) or vector containing a human *CDH1* (*FOXA2*^{KD}/Lenti-*CDH1* HEC-1-B) cDNA (OriGene) per the manufacturer's instructions.

Endometrial organoids

a) *<u>Culture and expansion</u>*

Endometrial organoids were derived from 16-20 week old *BAC-Sprr2f-Cre*; *Foxa2*$^{L/L}$, *BAC-Sprr2f-Cre*; *Pten*$^{L/L}$ or *BAC-Sprr2f-Cre*; *Foxa2*$^{L/L}$, *Pten*$^{L/L}$ mice. The uteri were washed in PBS, cut into small pieces (2-3 mm) using sterile scalpels and digested in Collagenase V/Dispase II enzymatic solution (0.4 mg/ml Collagenase V and 1.25 U/ml Dispase II: Sigma) in Advanced DMEM/F12 (Thermo Fisher Scientific) at 37^{0}C for 1 hr with intermittent shaking. The digestion mixture was triturated several times to disrupt epithelial glands into single cells. Tissue digestion was stopped by adding Advanced DMEM/F12 containing 10% FBS. Isolated cells were pelleted, resuspended in 98 µl FACS buffer (HBSS + 3% FBS) + 2 µl of FCR blocking buffer (Miltenyi Biotec) and incubated at 4°C for 10 min. After blocking, cells were pelleted and incubated with EpCAM antibody (1:40 dilution, BD Biosciences) at 4°C for 1 hr. Cells were washed, stained with propidium iodide (0.5 µg/ml, BD Biosciences) and used for flow sorting of EpCAM^{+ve}/PI^{-ve} live epithelial cells. FACS sorted epithelial cells were resuspended in Matrigel (Corning) and 20 µl of Matrigel-cell suspension (500 cells in 20 µl Matrigel) was plated in individual wells of a 48-well

plate and incubated at 37^{0}C for 5 min to solidify. The dome containing cells was overlaid with 250 µl of organoid expansion medium (Advanced DMEM/F12 supplemented with pen/strep [1%], Glutamax [2mM, Thermo Fisher Scientific], insulin-transferrin-selenium [1%, Thermo Fisher Scientific], nicotinamide [1mM, Sigma], N-2 supplement [1%, Thermo Fisher Scientific], B-27 supplement [2%, Thermo Fisher Scientific], R-Spondin1 [100 ng/ml, Peprotech], Noggin [100 ng/ml, Peprotech], FGF10 [100 ng/ml, R&D Systems], EGF [50 ng/ml, Peprotech] and Wnt3a [50 ng/ml, Peprotech]) to form organoids with media changed every 3 days.

b) Fixation and staining

For immunostaining, the day 12 organoids were harvested from Matrigel by scraping with a pipette tip, washed in PBS, and fixed in 4% PFA (Electron Microscopy Sciences) for 20 min at room temperature (RT). Following fixation, organoids were gently pelleted and overlaid with 200 µl of low melting agarose (Lonza) at 42^{0}C and permitted to solidify. The agarose plugs containing organoids were embedded in paraffin and 5 µm sections were prepared. The sections were deparaffinized in xylene followed by rehydration through a series of graded ethanol. Antigen retrieval was performed using sodium citrate buffer (pH 6.0, Vector Laboratories). Slides were washed with TBS containing 0.1% Tween 20 and incubated with cytokeratin, E-cadherin and GM130 antibodies (details in the resources table) overnight at 4°C. Sections were washed and incubated with Alexa Fluor secondary antibodies for 1 hr at RT and cover slipped using VECTASHIELD antifade mounting media with DAPI (Vector Laboratories). Images were taken on a Nikon ECLIPSE Ni fluorescence microscope. For 3D z-stack imaging, whole-mount organoids were fixed, permeabilized and stained with antibodies followed by imaging on a Zeiss LSM880 inverted confocal microscope.

c) Proliferation assays

Organoids were harvested from Matrigel using cell recovery solution (Corning) and trypsinized in TrypLE express enzyme (Thermo Fisher Scientific). The cells were resuspended in a 15 µl

Matrigel drop containing 500 cells in the wells of a 96-well plate in triplicate, overlaid with 150 µl of organoid expansion medium and incubated at 37°C containing 5% CO_2. The luminescence signal was recorded with a Tecan Spark 10M microplate reader on day 3, 5, 7 and 10 with Cell Titer-Glo 3D reagent (Promega).

Tumor grafting

All procedures for tumor xenograft mice were approved by the University of Texas Southwestern Medical Center Institutional Animal Care and Use Committee. Six- to eight-week-old female NOD *scid* gamma mice (Jackson Laboratory) were used. Ishikawa (ISK) or FP cells (1 X 10^6) +/- transduced with *FOXA2* lentiviral particles (OriGene, as described above) were injected subcutaneously into the flanks of the mice (n = 4/group) in 200 µL of sterile DPBS and Matrigel (1:1). Tumor dimensions were measured twice in a week by a digital caliper and volumes were calculated using the formula (length x $width^2$)/2. Tumor weights were determined at the end of the experiment.

Immunohistochemistry and quantification

Human EC TMA slides (US Biomax) were deparaffinized in xylene followed by rehydration in graded alcohols and water. Antigen retrieval was performed using sodium citrate buffer (pH 6.0, Vector Laboratories). Slides were washed with TBS containing 0.1% Tween 20 and incubated with 3% hydrogen peroxide (H_2O_2) in water for 15 min to block endogenous peroxidase. Sections were blocked with 2% BSA in PBS containing 0.1% Triton X-100 for 1 hr at RT. After blocking, tissue sections were incubated with primary antibodies against FOXA2, ERα or PR (details in the resources table) at 4^oC overnight. Slides were then washed and incubated with peroxidase-conjugated secondary antibodies (Vector Laboratories) and DAB substrate (Agilent Dako) to detect bound antibodies. Tissue sections were counterstained with hematoxylin. Images were captured using an Aperio AT2 slide scanner (Leica Biosystems) and analyzed using Aperio

ImageScope software. Immunohistochemistry intensity score (H-score) was calculated from pixel intensity values (the sum of 3 x % of pixels with strong staining + 2 x % of pixels with moderate staining + 1 x % pixels with weak staining).

Immunostaining

Ishikawa and HEC-1-B cells were cultured on glass slides, fixed in 4% paraformaldehyde (Electron Microscopy Sciences) for 20 min and processed for immunofluorescence. Cells were permeabilized in PBS containing 0.5% Triton X-100 for 10 min at 4^0C, rinsed in PBS/Glycine buffer (PBS containing 0.1 M Glycine), blocked in immunofluorescence buffer (130 mM NaCl, 7 mM Na_2HPO_4, 3.5 mM NaH_2PO_4, 0.1% BSA, 0.2% Triton X-100, 0.05% Tween 20) containing 10% goat serum and incubated with primary antibodies overnight at 4 ^{0}C. Cells were then washed in TBS containing 0.1% Tween 20, incubated with Alexa Fluor secondary antibodies for 1 hr at RT and cover slipped using VECTASHIELD antifade mounting media with DAPI (Vector Laboratories). Images were captured on a Nikon ECLIPSE Ni fluorescence microscope using NIS-Elements BR software.

Western blots

EC cell lines were lysed in ice-cold RIPA buffer (Thermo Fisher Scientific) containing protease and phosphatase inhibitors (Sigma). Following lysis, the protein extracts were centrifuged at 12,000 rpm for 5 min at 4^0C and supernatant was collected. 20 µg of protein was mixed with 4X NuPAGE LDS Sample Buffer (Thermo Fisher Scientific), incubated at 95^0C for 5 min and resolved by 4-12% NuPAGE gels (Thermo Fisher Scientific). Transfer to PVDF membrane (Sigma) was with the Trans-Blot Turbo Transfer System (Bio-Rad Labs). The membrane was blocked in TBS-T (0.1% Tween 20 in TBS) containing 5% skim milk (w/v) for 1 hr at RT and probed with primary antibody at 4^0C overnight. The membrane was washed and incubated with horseradish peroxidase (HRP) conjugated secondary antibody (Amersham) for 1

hr at RT and developed using SuperSignal West Dura Extended Duration Substrate (Thermo Fisher Scientific) for detection of HRP and the protein bands were detected by chemiluminescence using a ChemiDoc Imaging System (Bio-Rad Labs). β-Actin or GAPDH were used as loading controls.

Total RNA isolation, cDNA synthesis and qRT-PCR

Total RNA was isolated from EC cell lines using the RNeasy Plus Mini Kit (Qiagen) following the manufacturer's instructions. cDNA was synthesized from 1 μg of total RNA using SuperScript VILO master mix (Thermo Fisher Scientific). The cDNA was amplified using sequence specific primers (see resources table below). Quantitative real-time PCR (qRT-PCR) was performed using RT^2 SYBR Green ROX qPCR Mastermix (Qiagen) on a QuantStudio Real-Time PCR System (Applied Biosystems) through a pre-incubation step, and 40 amplification cycles. The comparative Ct ($2^{-\Delta\Delta Ct}$) method was used to compare the expression level of the target gene with the housekeeping gene (*GAPDH*) in different groups.

Library preparation, RNA seq and data analysis

Total RNA samples were quantified by Qubit fluorometer and submitted to the McDermott Next Generation Sequencing Core at UT Southwestern for library preparation. 1 μg of DNase treated high quality RNA (RIN score ≥ 8 per Agilent 2100 Bioanalyzer) was prepared with the TruSeq Stranded Total RNA LT Sample Prep Kit (Illumina). Total RNA was depleted of its rRNA and fragmented before strand-specific cDNA synthesis. cDNA was poly-A tailed and indexed adapters were ligated. After adapter ligation, samples were PCR amplified, purified with Ampure XP beads, and validated again on the Agilent 2100 Bioanalyzer. Samples were Qubit quantified before being normalized and run on the Illumina HiSeq 2500 using SBS v3 reagents with a read configuration of 75 bp, single end reads. 25-35 million reads were generated per sample.

The Fastq files were subjected to quality check using fastqc (version 0.11.2, http://www.bioinformatics.babraham.ac.uk/projects/fastqc) and fastq_screen (version 0.4.4, http://www.bioinformatics.babraham.ac.uk/projects/ fastq_screen). Fastq files were mapped to Homo Sapiens (hg19, UCSC) and Mus musculus reference genomes (mm10, UCSC) using STAR (1). Gene quantification was performed using featureCounts (2) and the differential expression analysis was performed using edgeR (3).

Only genes with log_2CPM >0 and log_2FC >1 were considered. Different significance levels were used to define differentially expressed genes depending on the scenarios. Pathway analysis was performed using webgestalt (http://www.webgestalt.org/). The threshold for enriched biological processes in the gene ontology was set to FDR <0.05. The dot plots were generated using ggplot2 (https://ggplot2.tidyverse.org/reference/geom_dotplot.html).

Chromatin Immunoprecipitation (ChIP) and data analysis

The chromatin that was bound by acetylated Histone in ISK-EV and ISK-*FOXA2* cells was analyzed. Crosslinking was carried out using 1% formaldehyde at RT. Formaldehyde was quenched with 1.25 M glycine. Cells were washed with PBS, lysed, sonicated, and the chromatin was used in a ChIP reaction using Histone H3 acetyl Lys27 antibody and HighCell# ChIP kit protein G (Diagenode) per the manufacturer's recommendation (4). The ChIP DNA was reverse crosslinked, eluted and purified (5), and used to prepare next generation sequencing libraries (KAPA Hyper Prep, KAPA Biosystems, MA). The ChIP sequencing (ChIP-Seq) libraries, after appropriate size selection using AMPure XP Beads (Beckman Coulter Sciences, IN) were sequenced with a read-length of 75bp at the UTSW McDermott Sequencing Core. The quality of the raw data was checked using fastqc (v0.11.8) (6). Raw reads were mapped to hg19 using BWA (v 0.7.17-r1188) (7). Low quality and duplicated reads were removed using Picard (2.20.3, http://broadinstitute.github.io/picard). Peak calling was performed by MACS2 (v 2.1.0) (8) using

default parameters with input DNA as negative control. Deeptools (2.4.2) (9) was used to plot average coverage figures and heatmaps. The transcription start site (TSS) positions were obtained from UCSC table browser (https://genome.ucsc.edu/cgi-bin/hgTables). Integrated Genomics Viewer (IGV, v2.8.0) (10) was used to visualize the ChIP-seq peak signals.

Cell cycle analysis

Ishikawa (ISK) and FP cells (70-80% confluent) transfected with empty vector (ISK-EV and FP-EV) and transduced with FOXA2 lentiviral particles (ISK-*FOXA2* and FP-*Foxa2*) were fixed in 2% paraformaldehyde solution for 10 min at RT. After fixation cells were washed in PBS and permeabilized in PBS containing 0.1% Triton X-100 for 15 min at RT. Lysates were passed through a 40 µm nylon cell strainer (Fisher Scientific) to remove cell debris, followed by incubated with 50 µg/mL of propidium iodide (PI, BD Biosciences) to stain DNA and 100 µg/mL of RNaseA to remove excess RNA. A total of 1 x 10^6 nuclei from the total population were analyzed on a FACS analyzer with 488 nm LASER line to detect PI-stained nuclei. The percentage of cells within the G0/G1, S and G2/M phases of the cell cycle were determined by analyzing the histogram output using FlowJo analysis software.

IncuCyte cell proliferation and scratch assay

The IncuCyte live-cell imaging and analysis system was used for cell proliferation and wound closure assays. *FOXA2* +/- Ishikawa and FP cells (5,000 cells/each well of flat bottom 96-well plate) were grown to 90-100% confluency with images acquired every 6 hrs. Cell proliferation curves were determined with IncuCyte software by plotting normalized confluency (ratio of confluency at each 6 hr interval to confluency at time 0).

For wound closure assays, Ishikawa cells were cultured to reach 90-100% confluency and a single homogenous scratch per well of 96-well plate was made with the WoundMaker tool (Essen Bioscience). Cells were washed twice and replaced with complete medium. Images of migrating

cells near the wound were taken every 4 hrs and wound width (distance between leading edges of each cell front) was calculated with the IncuCyte software.

Cell adhesion assays

FOXA2 +/- Ishikawa cells (150,000) were seeded onto MicroMatrix ECM array slide (Advanced BioMatrix) and allowed to adhere for 16 hrs. Following cell attachment, the slide was washed in DPBS to remove unbound cells. The cells adhered to micromatrix spots after the wash were captured and counted per the manufacturer's instructions.

Transwell migration and invasion assays

HEC-1-B cells (control and *FOXA2* knockdown) were grown till 70-80% confluency and serum starved for 4 hrs. For migration assays, 1×10^5 cells in serum-free medium were placed in the upper chamber of 24-well Transwell inserts (8-μm pore size, Fisher Scientific) and complete medium containing 10% FBS was added to the lower chamber. For invasion assays, inserts were coated with Matrigel and DPBS (20 μL Matrigel + 100 μL DPBS), incubated at 37^0C for 2 hrs and then dried at RT overnight. Cells in serum-free medium were added to the upper chamber of the insert and medium containing 10% FBS was added to the lower chamber. After 16 hrs, migrated or invaded cells on the insert membrane were fixed in 70% alcohol and stained with crystal violet (0.5%) for 10 min. Cells in the inner chamber were removed with cotton swabs. Images of migrated or invaded cells attached to the membrane were quantified with ImageJ (NIH, USA).

Resources

Antibodies	Source	Identifier	Dilution
Rabbit pAb anti-FOXA1	Millipore Sigma	Cat #HPA050505; RRID: AB_2681148	IHC: 1:500; WB: 1:1000
Rabbit mAb anti-FOXA2 (EPR4466)	Abcam	Cat #ab108422; RRID: AB_11157157	IHC: 1:500; IF: 1:500; WB: 1:1000
Rabbit pAb anti-H3K27ac	Active Motif	Cat #39134; RRID: AB_2722569	2 µg for 25 µg chromatin/ChIP
Rabbit mAb anti-Acetyl-Histone H3 (Lys27) (D5E4)	Cell Signaling Technology	Cat #8173; RRID: AB_10949503	WB: 1:500
Rabbit mAb anti-Histone H3 (D1H2)	Cell Signaling Technology	Cat #4499; RRID: AB_10544537	WB: 1:1000
Rabbit mAb anti-Estrogen Receptor α (D6R2W)	Cell Signaling Technology	Cat #13258; RRID: AB_2632959	IHC: 1:500
Rabbit mAb anti-Progesterone Receptor A/B (D8Q2J)	Cell Signaling Technology	Cat #8757; RRID: AB_2797144	IHC: 1:500
Rabbit mAb anti-c-Myc (Y69)	Abcam	Cat #ab32072; RRID: AB_731658	WB: 1:1000
Rabbit mAb anti-E-Cadherin (24E10)	Cell Signaling Technology	Cat #3195; RRID: AB_2291471	IF: 1:200; WB: 1:1000
Mouse mAb anti-Vimentin (VI-10)	Abcam	Cat #ab20346; RRID: AB_445527	IF: 1:200; WB: 1:1000
Rabbit mAb anti-β-Catenin (D10A8)	Cell Signaling Technology	Cat #8480; RRID: AB_11127855	IF: 1:200; WB: 1:1000
Rabbit pAb anti-Snail + Slug	Abcam	Cat #ab180714; RRID: AB_2728773	WB: 1:1000
Mouse mAb anti-SIP1/ZEB2 (E-11)	Santa Cruz Biotechnology	Cat #sc-271984; RRID: AB_10708399	WB: 1:1000
Rabbit mAb anti-Cyclin A2 (E1D9T)	Cell Signaling Technology	Cat #91500; RRID: N/A	WB: 1:1000
Rabbit Ab anti-Cyclin B1	Cell Signaling Technology	Cat #4138; RRID: AB_2072132	WB: 1:1000
Rabbit mAb anti-Cyclin D1 (SP4)	Abcam	Cat #ab16663; RRID: AB_443423	WB: 1:1000
Rabbit mAb anti-Cyclin E1 (D7T3U)	Cell Signaling Technology	Cat #20808; RRID: AB_2783554	WB: 1:1000
Rabbit mAb anti-GAPDH (14C10)	Cell Signaling Technology	Cat #2118; RRID: AB_561053	WB: 1:4000
Mouse mAb anti-β-Actin (8H10D10)	Cell Signaling Technology	Cat #3700; RRID: AB_2242334	WB: 1:4000
Mouse mAb anti-GM130 (Clone 35)	BD Biosciences	Cat #610822; RRID: AB_398141	IF: 1:100
Rabbit pAb anti-Cytokeratin	Abcam	Cat #ab9377; RRID: AB_307222	IF: 1:200
Rabbit mAb anti-PTEN (138G6)	Cell Signaling Technology	Cat #9559; RRID: AB_390810	IF: 1:200
Phalloidin-TRITC	Millipore Sigma	Cat #P1951; RRID: AB_2315148	IF: 1:400
Phalloidin-FITC	Millipore Sigma	Cat #P5282; RRID: N/A	IF: 1:400
Mouse Ab anti-CD326 (Ep-CAM) (Clone G8.8)	BD Biosciences	Cat #563214; RRID: AB_2738073	FACS: 1:40
Donkey anti-rabbit IgG, HRP linked Ab	Amersham	Cat #NA934; RRID: AB_772206	WB: 1:1000
Sheep anti-mouse IgG, HRP linked Ab	Amersham	Cat #NA931; RRID: AB_772210	WB: 1:1000
Alexa Fluor 555 goat anti-rabbit IgG (H+L)	Thermo Fisher Scientific	Cat #A-21429; RRID: AB_2535850	IF: 1:250

Alexa Fluor 555 goat anti-mouse IgG (H+L)	Thermo Fisher Scientific	Cat #A-21424; RRID: AB_141780	IF: 1:250
Alexa Fluor 488 goat anti-rabbit IgG (H+L)	Thermo Fisher Scientific	Cat #A-11008; RRID: AB_143165	IF: 1:250
ImmPRESS HRP horse anti-rabbit IgG	Vector Laboratories	Cat #MP-7401; RRID: AB_2336529	N/A

**Reagents**	**Source**	**Identifier**
Collagenase V	Millipore Sigma	Cat #C9263; CAS #9001-12-1
Dispase II	Millipore Sigma	Cat #D4693; CAS #42613-33-2
FCR blocking buffer	Miltenyi Biotec	Cat #130-092-575
Propidium Iodide staining solution	BD Biosciences	Cat #556463
Advanced DMEM/F12	Thermo Fisher Scientific	Cat #12634010
HBSS	Thermo Fisher Scientific	Cat #14170120
Fetal Bovine Serum	Millipore Sigma	Cat #F4135
Penicillin-streptomycin	Thermo Fisher Scientific	Cat #15240062
Glutamax	Thermo Fisher Scientific	Cat #35050061
Insulin-Transferrin-Selenium (ITS)	Thermo Fisher Scientific	Cat #41400-045
Nicotinamide	Millipore Sigma	Cat #N0636; CAS #98-92-0
N-2 supplement	Thermo Fisher Scientific	Cat #17502048
B-27 supplement	Thermo Fisher Scientific	Cat #12587010
Recombinant murine R-Spondin-1	Peprotech	Cat #315-32
Recombinant murine Noggin	Peprotech	Cat #250-38
Recombinant murine EGF	Peprotech	Cat #315-09
Recombinant murine Wnt-3a	Peprotech	Cat #315-20
Recombinant mouse FGF-10	R&D Systems	Cat #6224
Matrigel	Corning	Cat #356231
Cell recovery solution	Corning	Cat #354253
TrypLE Express Enzyme	Thermo Fisher Scientific	Cat #12604021
Puromycin dihydrochloride	Thermo Fisher Scientific	Cat #A1113803
Hexadimethrine bromide (Polybrene)	Millipore Sigma	Cat #H9268; CAS #28728-55-4
Paraformaldehyde 16% solution EM grade	Electron Microscopy Sciences	Cat #15710
Sea Plaque low melting agarose	Lonza	Cat #50100
Antigen unmasking solution, Citrate-based	Vector Laboratories	Cat #H3300
Tween 20	Millipore Sigma	Cat #P1379; CAS #9005-64-5
TBS buffer	VWR Life Science	Cat #97064-338
Phalloidin, Fluorescein Isothiocyanate Labeled	Millipore Sigma	Cat #P5282
Vectashield Vibrance Antifade Mounting Media Medium with DAPI	Fisher Scientific	Cat #H-1800
RIPA lysis and extraction buffer	Thermo Fisher Scientific	Cat #89900
Protease inhibitor cocktail	Millipore Sigma	Cat #P8340
Phosphatase inhibitor cocktail	Millipore Sigma	Cat #P5726
NuPAGE LDS Sample Buffer	Thermo Fisher Scientific	Cat #NP0007
NuPAGE gels	Thermo Fisher Scientific	Cat #NP0321BOX
PVDF membrane	Millipore Sigma	Cat #IPVH00010
Hydrogen peroxide	Fisher Scientific	Cat #BP2633500; CAS #7722-84-1
SuperScript VILO master mix	Thermo Fisher Scientific	Cat #11755050
RT^2 SYBR green ROX qPCR master mix	Qiagen	Cat #330524

RNase A	Thermo Fisher Scientific	Cat #12091021
Cell strainer snap cap, 5 ml	Fisher Scientific	Cat #0877123
Cell culture inserts	Fisher Scientific	Cat #0877121
Endometrial cancer tissue array	US Biomax	Cat #EMC1021
ReproLife™ Reproductive Medium Complete Kit	Lifeline Cell Technology	Cat #LL-0068

**Cell lines**		
Homo sapiens	**Source**	**Identifier or Reference**
Ishikawa	Millipore Sigma	Cat #99040201
AN3CA	ATCC	Cat #HTB-111
HEC-1-A	ATCC	Cat #HTB-112
HEC-1-B	ATCC	Cat #HTB-113
RL95-2	ATCC	Cat #CRL-1671
KLE	ATCC	Cat #CRL-1622
EN	DSMZ	Cat #ACC 564
MFE-319	DSMZ	Cat #ACC 423
EFE-184	DSMZ	Cat #ACC 230
MFE-280	DSMZ	Cat #ACC 410
MFE-296	DSMZ	Cat #ACC 419
EI	Obtained from M. Takayama	PMID: 7669752
EJ	Obtained from M. Takayama	PMID: 12889855
Endometrial (Uterine) Epithelial Cells (HEuEC)	Lifeline Cell Technology	Cat #FC-0078
Mus musculus	**Source**	**Additional information**
FP	This paper	See 'Mouse EC cell line (FP) derivation and culture' in Methods

**Commercial Assays**	**Source**	**Identifier**
RNeasy Plus Mini Kit	Qiagen	Cat #74136
Mycosensor PCR assay kit	Agilent Genomics	Cat #302108
ECM Select Array Kit Ultra-36	Advanced Biomatrix	Cat #5170
Cell Titer-Glo 3D Cell Viability Assay	Promega	Cat #G9683
Liquid DAB+, 2-component system, Immunohistochemistry visualization	Agilent Dako	Cat #K346811-2
Super Signal West Dura Extended Duration Substrate	Thermo Fisher Scientific	Cat #34076
HighCell# ChIP kit protein G	Diagenode	Cat #C01010063

**Vectors**	**Source**	**Identifier**
FOXA2 shRNA (TCCATCAACAACCTCATGTCCTCGGAGCA)	OriGene	Cat #TL304478
Foxa2 (NM_010446) mouse tagged ORF clone lentiviral particle	OriGene	Cat #MR227354L3V
FOXA2 (NM_153675) human tagged ORF clone lentiviral particle	OriGene	Cat #RC211408L3V
E Cadherin (CDH1) (NM_004360) human tagged ORF clone lentiviral particle	OriGene	Cat #RC220731L1V
Mouse c-Myc/Myc Full-length Gene in Lentivector, pre-packaged lentiviral Particles	G&P Biosciences	Cat #LEV0011

pLenti-C-Myc-DDK-P2A-Puro lentiviral gene expression vector	OriGene	Cat #PS100092

**Sequence of qPCR primers**		
**Human**	**Forward (5' to 3')**	**Reverse (5' to 3')**
AGR2	ATTGGCAGAGCAGTTTGTCC	GAGCTGTATCTGCAGGTTCGT
CNTN1	GCCCATGACAAAGAAGAAGC	CGACATGATCCCAGGTGATT
FOXA2	GAGAAGAAATCCATAACACCCCC	TTCTTTCCCGTTTTCCTCCTTAT
GAPDH	GCCACATCGCTCAGACACCAT	GAAGGGGTCATTGATGGCAA
PCDHA1	CAACCCTGACTGGCGTTACT	GGATACTGTTGGCCACTGCT
**Mouse**	**Forward (5' to 3')**	**Reverse (5' to 3')**
Agr2	TGGACGAATGCCCACACAGT	AGTGATGTCTGCCCTCACCG
Cdh1	GTCTCCTCATGGCTTTGC	CTTTAGATGCCGCTTCAC
Cdh2	TGAAACGGCGGGATAAAGAG	GGCTCCACAGTATCTGGTTG
Cntn1	CCTCATTCCGATTCCTGAAC	TCCTTGAACTGCACCACAAT
Ctnnb1	ATTGATTCGAAACCTTGCCC	AGCTCCAGTACACCCTTCTA
Foxa2	AGCAGAGCCCCAACAAGA	AGAGAGAGTGGCGGATGGAG
Gapdh	CAACTACATGGTCTACATGTT	CTCGCTCCTGGAAGATG
Snai1	GTCGTCCTTCTCGTCCACC	GGCCTGGCACTGGTATCTC
Snai2	TCCCATTAGTGACGAAGA	CCCAGGCTCACATATTCC
Vim	CGGCTGCGAGAGAAATTGC	CCACTTTCCGTTCAAGGTCAAG
Zeb1	CCATACGAATGCCCGAACT	ACAACGGCTTGCACCACA
Zeb2	GAGCTTGACCACCGACTC	TTGCAGGACTGCCTTGAT

Supplemental References

1. Dobin A, Davis CA, Schlesinger F, Drenkow J, Zaleski C, Jha S, et al. STAR: ultrafast universal RNA-seq aligner. *Bioinformatics.* 2013;29(1):15-21.
2. Liao Y, Smyth GK, and Shi W. featureCounts: an efficient general purpose program for assigning sequence reads to genomic features. *Bioinformatics.* 2014;30(7):923-30.
3. Robinson MD, McCarthy DJ, and Smyth GK. edgeR: a Bioconductor package for differential expression analysis of digital gene expression data. *Bioinformatics.* 2010;26(1):139-40.
4. Ramanand SG, Chen Y, Yuan J, Daescu K, Lambros MB, Houlahan KE, et al. The landscape of RNA polymerase II-associated chromatin interactions in prostate cancer. *J Clin Invest.* 2020;130(8):3987-4005.
5. Cotney JL, and Noonan JP. Chromatin immunoprecipitation with fixed animal tissues and preparation for high-throughput sequencing. *Cold Spring Harb Protoc.* 2015;2015(2):191-9.
6. Andrews S. FastQC. http://www.bioinformatics.babraham.ac.uk/projects/fastqc/.
7. Li H, and Durbin R. Fast and accurate short read alignment with Burrows-Wheeler transform. *Bioinformatics.* 2009;25(14):1754-60.
8. Zhang Y, Liu T, Meyer CA, Eeckhoute J, Johnson DS, Bernstein BE, et al. Model-based analysis of ChIP-Seq (MACS). *Genome Biol.* 2008;9(9):R137.
9. Ramirez F, Dundar F, Diehl S, Gruning BA, and Manke T. deepTools: a flexible platform for exploring deep-sequencing data. *Nucleic Acids Res.* 2014;42(Web Server issue):W187-91.
10. Robinson JT, Thorvaldsdottir H, Winckler W, Guttman M, Lander ES, Getz G, et al. Integrative genomics viewer. *Nat Biotechnol.* 2011;29(1):24-6.

Supplemental Figures and Legends

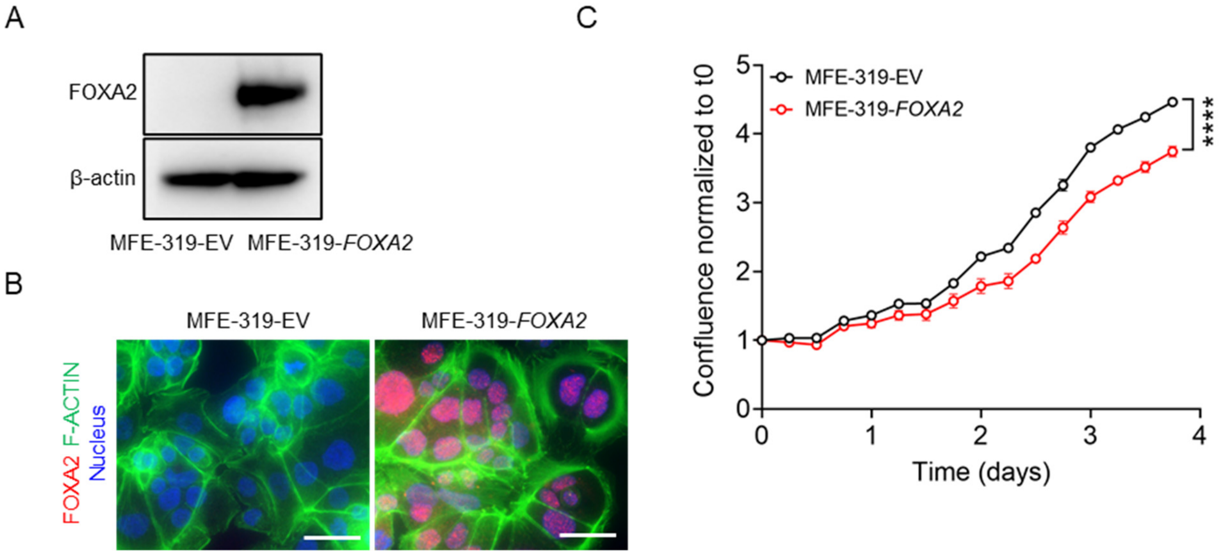

Supplemental Figure 1. FOXA2 re-expression suppresses MFE-319 cell proliferation.

(A) Western blot shows enforced FOXA2 expression following lentiviral transduction in MFE-319 cells (MFE-319-*FOXA2*). For negative controls, cells were subjected to lentiviral transduction with empty vector.

(B) Immunofluorescence of FOXA2 (red) and actin (green) in MFE-319-EV and MFE-319-*FOXA2* cells confirms expected nuclear localization in the latter. Nuclei were stained with DAPI (blue). Scale bars, 50 µm.

(C) Cell proliferation assay by live cell imaging of MFE-319-EV and MFE-319-*FOXA2* cells showing growth suppression by *FOXA2*. Percent confluency was normalized to time 0 (n=3). Data represents mean ± SEM; ****, P <0.0001, *t*-test.

A

Enriched GO pathways in up-regulated genes

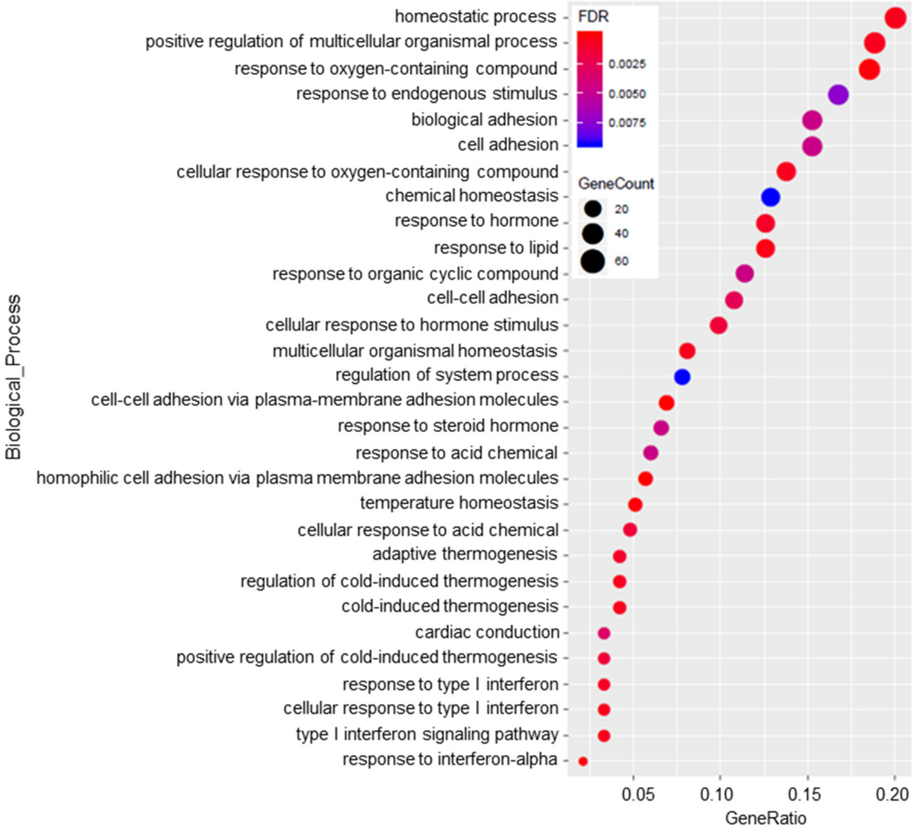

B

Enriched GO pathways in down-regulated genes

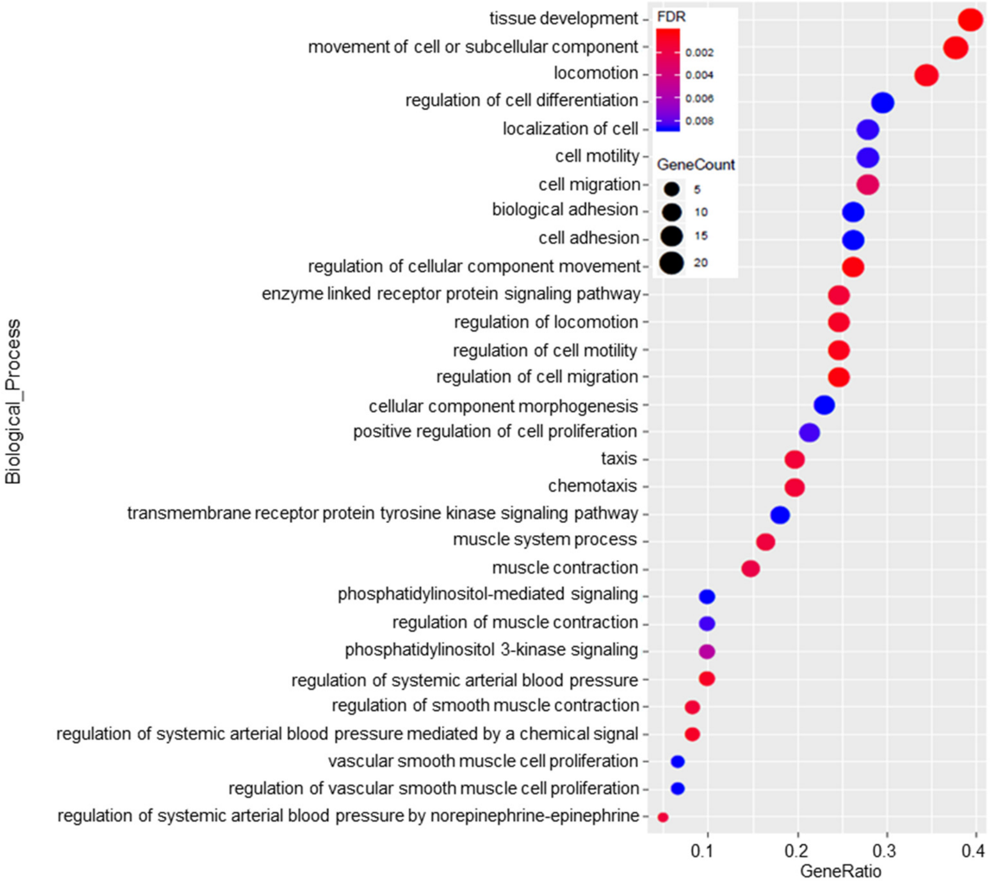

Supplemental Figure 2. Enriched Gene Ontology (GO) pathways in ISK-*FOXA2* cells.

(A and B) The dot plots show the identified GO biological pathways. GO analysis was performed on significantly up- and downregulated genes by RNA-seq (fold change > 2, P <0.0001, FDR <0.0005). The size of the dots represents the number of the genes associated with the GO term and the color of the dots represents the FDR values as shown in the inset.

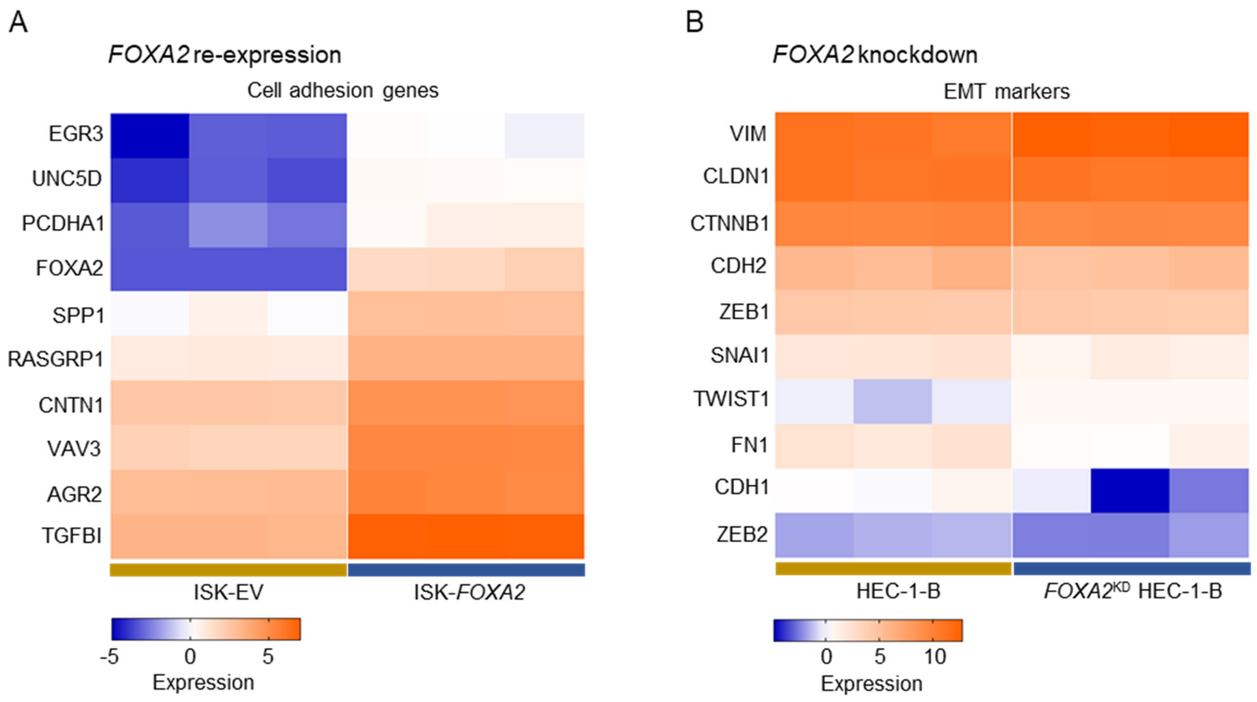

Supplemental Figure 3. Heatmap of differentially expressed cell adhesion and EMT genes in FOXA2 re-expressing and knockdown cells.

(A) Heatmap shows differentially expressed cell adhesion genes after *FOXA2* expression in Ishikawa (ISK) cells.

(B) Heatmap shows differentially expressed EMT genes in *FOXA2* knockdown HEC-1-B cells.

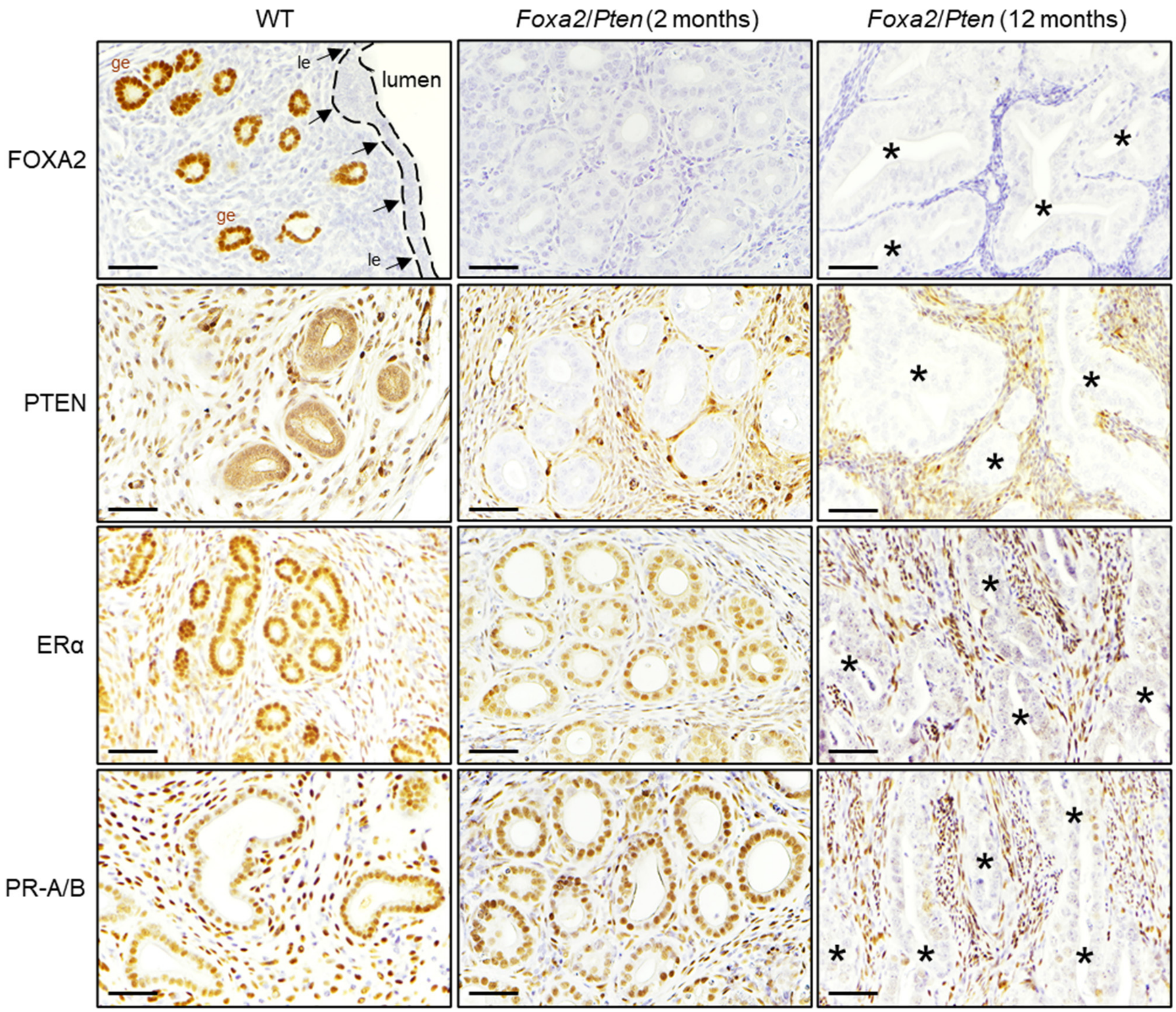

Supplemental Figure 4. Confirmation of FOXA2 and PTEN loss along with ERα and PR-A/B in *Foxa2*/*Pten* mouse uteri.

IHC panel shows expression patterns of FOXA2, PTEN, ERα and PR-A/B in *Foxa2*/*Pten* mouse uteri at 2 and 12 months of age compared to wild-type (WT) mouse. Black dashed line shows luminal epithelium in WT mouse. Tumor cells are marked by an asterisk. le, luminal epithelium; ge, glandular epithelium, scale bars, 50 μm.

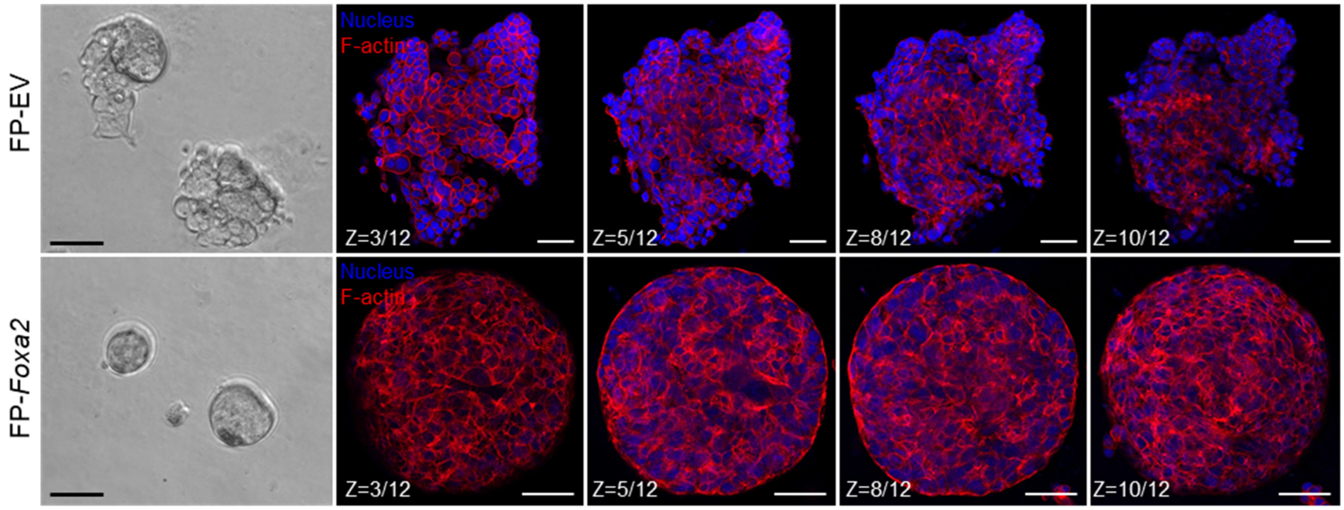

Supplemental Figure 5. Confocal z-stack images of FP and FP-*Foxa2* organoids.

Brightfield images of day 5 FP-EV and FP-*Foxa2* organoids. Organoids were stained with actin (red) and counterstained with DAPI (blue). Confocal z-stack images of 2μm thickness at indicated slices are shown. Scale bars, 50 μm.

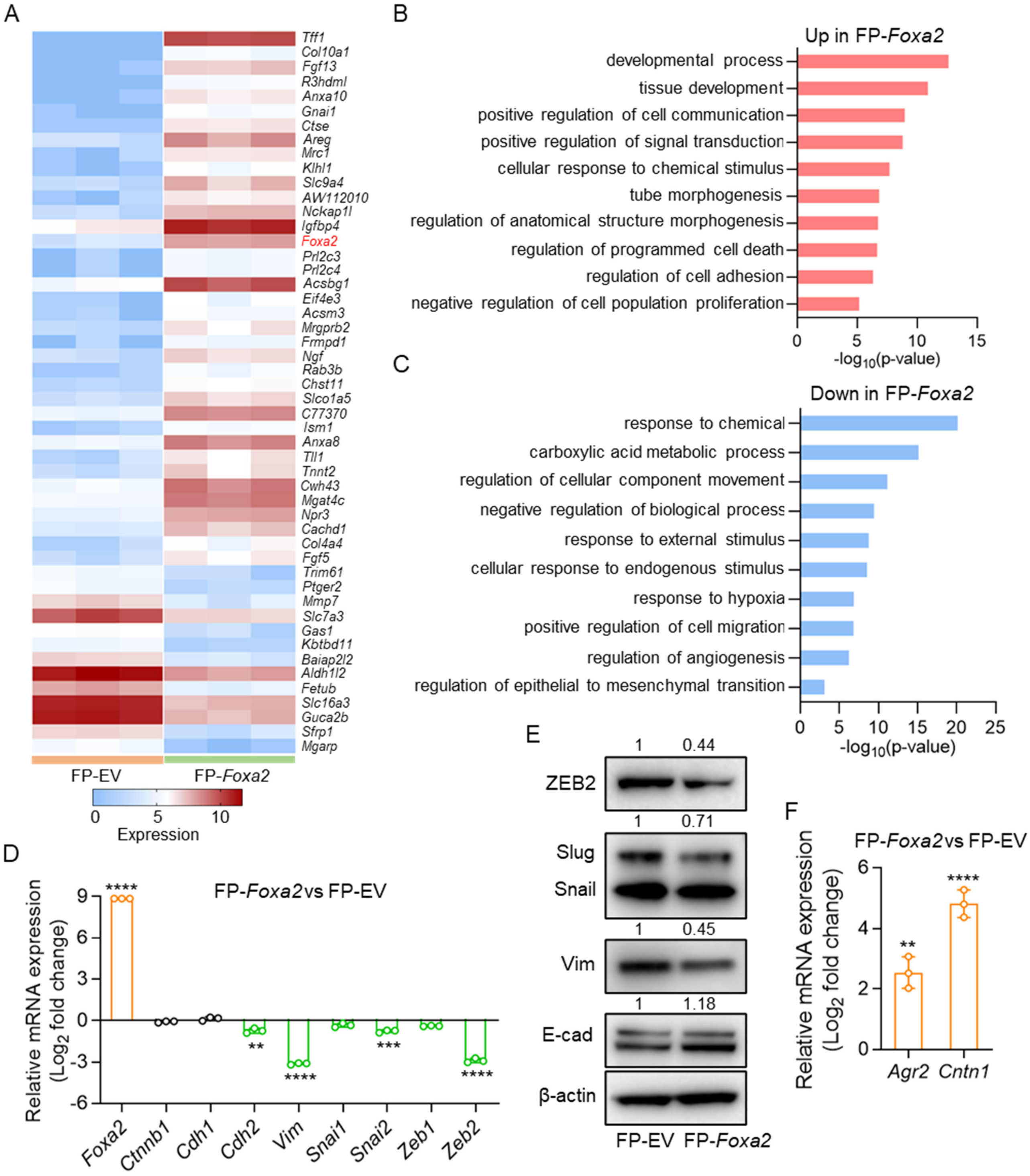
A
Tff1
Col10a1
Fgf13
R3hdml
Anxa10
Gnai1
Ctse
Areg
Mrc1
Klhl1
Slc9a4
AW112010
Nckap1l
Igfbp4
Foxa2
Prl2c3
Prl2c4
Acsbg1
Eif4e3
Acsm3
Mrgprb2
Frmpd1
Ngf
Rab3b
Chst11
Slco1a5
C77370
Ism1
Anxa8
Tll1
Tnnt2
Cwh43
Mgat4c
Npr3
Cachd1
Col4a4
Fgf5
Trim61
Ptger2
Mmp7
Slc7a3
Gas1
Kbtbd11
Baiap2l2
Aldh1l2
Fetub
Slc16a3
Guca2b
Sfrp1
Mgarp
FP-EV
FP-Foxa2
0
5
10
Expression
B
Up in FP-Foxa2
developmental process
tissue development
positive regulation of cell communication
positive regulation of signal transduction
cellular response to chemical stimulus
tube morphogenesis
regulation of anatomical structure morphogenesis
regulation of programmed cell death
regulation of cell adhesion
negative regulation of cell population proliferation
0
5
10
15
-log10(p-value)
C
Down in FP-Foxa2
response to chemical
carboxylic acid metabolic process
regulation of cellular component movement
negative regulation of biological process
response to external stimulus
cellular response to endogenous stimulus
response to hypoxia
positive regulation of cell migration
regulation of angiogenesis
regulation of epithelial to mesenchymal transition
0
5
10
15
20
25
-log10(p-value)
D
FP-Foxa2 vs FP-EV
Relative mRNA expression
(Log2 fold change)
9
6
3
0
-3
-6
****
**
****
***
****
Foxa2
Ctnnb1
Cdh1
Cdh2
Vim
Snai1
Snai2
Zeb1
Zeb2
E
1
0.44
ZEB2
1
0.71
Slug
Snail
1
0.45
Vim
1
1.18
E-cad
β-actin
FP-EV
FP-Foxa2
F
FP-Foxa2 vs FP-EV
Relative mRNA expression
(Log2 fold change)
6
4
2
0
**
****
Agr2
Cntn1

Supplemental Figure 6. *Foxa2* re-expression suppresses EMT.

(A) Heatmap showing top 50 differentially expressed genes between FP-EV and FP-*Foxa2* cells.

(B and C) Enriched up- and downregulated GO pathways in FP-*Foxa2* cells. GO analysis was performed with the significantly up- and downregulated genes by RNA-seq (fold change >2, P <0.0001, Log_2CPM >0).

(D) Relative fold-change in expression of different EMT markers in FP-*Foxa2* vs FP-EV cells (n=3). Data represents mean ± SEM; **, P <0.01; ***, P <0.001; ****, P <0.0001, multiple *t*-tests.

(E) Protein expression by western blot in FP-EV and FP-*Foxa2* cells.

(F) Relative fold change expression of cell adhesion genes (*Agr2* and *Cntn1*) in FP-*Foxa2* vs FP-EV cells (n=3). Data represents mean ± SEM; **, P <0.01; ****, P <0.0001, multiple *t*-tests.

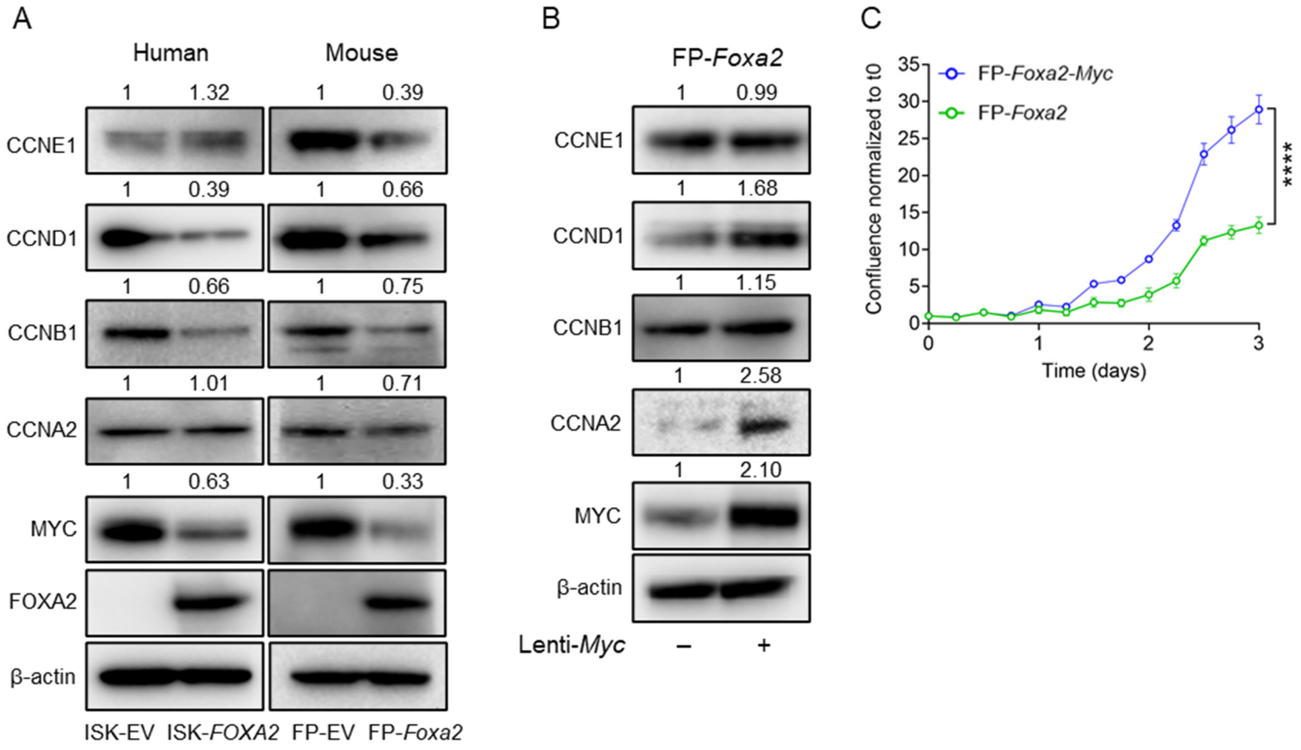

Supplemental Figure 7. *MYC* is negatively regulated by FOXA2, with functional consequences.

(A) MYC, and *MYC* targeted cell cycle checkpoint protein expression in human (ISK-*FOXA2*) and mouse (FP-*Foxa2*) EC cell lines by western blot.

(B) Expression of *Myc* targeted cell cycle checkpoint proteins in FP-*Foxa2* cells after lentiviral transduction of *Myc*.

(C) Growth comparison of FP-*Foxa2* and FP-*Foxa2*-*Myc* cells by IncuCyte real-time live cell imaging (n=3). Error bars represent mean ± SEM; ****, *P* <0.0001, *t*-test.

Supplemental Movie 1 (*see separate Supplemental Movie File*). Movie shows confocal z-stack images of 2µm thick slices of FP-EV and FP-Foxa2 organoids. Organoids were stained with actin (red) and counterstained with DAPI (blue).

Supplemental Tables (*see separate Supplemental Table File*)

Supplemental Table 1. List of differentially expressed genes (P < 0.0001, FC > 2, Log2CPM > 0) in ISK-FOXA2 vs ISK cells. FC, fold change; CPM, counts per million reads mapped; LR, likelihood ratio, FDR, false discovery rate

Supplemental Table 2. List of component genes in up- and down-regulated gene ontology (GO) catergories.

Supplemental Table 3. List of differentially expressed genes (FDR < 0.01, FC > 2, Log2CPM > 0) in FOXA2KD HEC1B vs HEC1B cells. FC, fold change; CPM, counts per million reads mapped; LR, likelihood ratio, FDR, false discovery rate

Supplemental Table 4. List of differentially expressed genes (P < 0.001, FC > 2, Log2CPM > 0) in FP-Foxa2 vs FP cells. FC, fold change; CPM, counts per million reads mapped; LR, likelihood ratio, FDR, false discovery rate
